# A flexible mixed-optimization with *H*_∞_ control for coupled twin rotor MIMO system based on the method of inequality (MOI)- An experimental study

**DOI:** 10.1371/journal.pone.0300305

**Published:** 2024-03-22

**Authors:** Nadir Abbas, Xiaodong Liu, Jamshed Iqbal

**Affiliations:** 1 Faculty of Electronic Information and Electrical Engineering, Dalian University of Technology, Dalian, China; 2 School of Computer Science, Faculty of Science and Engineering, University of Hull, Hull, United Kingdom; Osaka University, JAPAN

## Abstract

This article introduces a cutting-edge *H*_∞_ model-based control method for uncertain Multi Input Multi Output (MIMO) systems, specifically focusing on UAVs, through a flexible mixed-optimization framework using the Method of Inequality (MOI). The proposed approach adaptively addresses crucial challenges such as unmodeled dynamics, noise interference, and parameter variations. Central to the design is a two-step controller development process. The first step involves Nonlinear Dynamic Inversion (NDI) and system decoupling for simplification, while the second step integrates *H*_∞_ control with MOI for optimal response tuning. This strategy is distinguished by its adaptability and focus on balancing robust stability and performance, effectively managing the intricate cross-coupling dynamics in UAV systems. The effectiveness of the proposed approach is validated through simulations conducted in MATLAB/Simulink environment. Results demonstrated the efficiency of the proposed robust control approach as evidenced by reduced steady-state error, diminished overshoot, and faster system response times, thus significantly outperforming traditional control methods.

## 1 Introduction

Nonlinearity plays a crucial role in unmanned aerial vehicles (UAVs) and their operations, particularly in modern warfare scenarios. Nonlinearities refer to complex and often unpredictable behaviors that arise from nonlinear relationships between input and output signals or states of a system [1]. In UAVs, nonlinearities can arise from a range of factors, such as the aerodynamic properties of the vehicle, the dynamics of its propulsion and control systems, and the effects of external disturbances like wind and turbulence in [2]. These nonlinearities can make UAVs more challenging to model and control than linear systems, but they also offer several advantages in terms of their capabilities and performance.

An essential advantage of nonlinear UAVs lies in their capacity to function within intricate, ever-changing settings. Utilizing linear control algorithms empowers UAVs to adapt swiftly to shifting circumstances and promptly react to unforeseen disruptions. This capability allows them to efficiently traverse cluttered or congested environments, evade obstacles, and precisely track moving targets [3–6]. Nonlinear UAVs provide numerous merits in this context, including their adeptness at operating in jammed environments, their resilience against detection and interception, and their capability to execute intricate maneuvers while maintaining high speeds with heightened stability and precision. The significance of nonlinear characteristics in UAVs underscores their growing importance in the contemporary landscape, where advanced control and navigation systems are required to facilitate their effective operation in intricate and unpredictable surroundings [7–9]. The nomenclature for a UAV, including all pertinent parameters and their corresponding symbols, is comprehensively detailed in Table 1.

**Table 1:** Nomenclature.

Symbol	Description	Symbol	Description
*θ*	angular displacement of the main rotor	$\dot{\theta }$	angular velocity of the main rotor
*φ*	angular displacement of the tail rotor	$\dot{\varphi }$	angular velocity of the tail rotor.
*a*_1_, *a*_2_	constants related to the main and tail rotors	*b*_1_, *b*_2_	constants related to the main and tail rotors.
*I*_1_, *I*_2_	moment of inertia of the main and tail rotors, respectively	*τ*_1_, *τ*_2_	input control signals applied to the main and tail rotors, respectively.
*M*_*g*_	torque produced by the tail rotor (control input)	$k_{g_{y}}$	constant related to the tail rotor
*x* ∈ *R*	real number of states	*u* ∈ *R*	input signal
*C*_*c*_	controllability matrix	*O*_*o*_	observability matrix
*A*	system matrix	*α*	order of the matrix
*u*_*dis*_	disturbance	*J*	jacobian matrix
*B*_1*θ*_ *B*_2*θ*_ *B*_1*φ*_ *B*_2*φ*_	frictional parameters	*k*_*gy*_	gyroscopic parameter
*k*_1_	gain of main motor	*k*_2_	gain tail rotor
*T*_11_, *T*_21_	constants of the main rotor	*T*_10_, *T*_20_	constants of the tail rotor
*u*_2_	y-axis control input force	*u*_1_	x-axis control input
*G*_*θ*_	vertical plane system	*G*_*ϕ*_	horizontal plane system
*e*_*z*_(*t*)	tracking of pitch and yaw angles.	*v*(*t*)	scaling factor
*G*	nonlinear system.	*r*	reference input
$\left(\Delta_{\tilde{M}},\Delta_{\tilde{N}}\right)$	unstructured perturbation	*G*_*d*_	disturbance of plant.
*T*	complementary sensitive function	*W*_1_, *W*_2_, *W*_*p*_, *W*_*u*_	weighted functions
*G*_*u*_	transfer matrix of the control signal	*K*_*y*_	feedback matrix functions
*K*_*r*_	transfer matrix of pre-filter	*d*	disturbance
Δ_*F*_	fictitious perturbation	*S*_*o*_	output sensitivity matrix
*S*_*o*_ = (*I* + *G*_*u*_*K*_*y*_) − 1	output sensitivity matrix function	$y_{c}=y+W_{n_{n}}$	output feedback vector
*S*_*i*_ = (*I* + *K*_*y*_*G*_*u*_) − 1	input sensitivity matrix function	*S*_*i*_	input sensitivity matrix
*w*_*p*_	performance matrix	*w*_*u*_	control action matrix
*w*_*m*_	model matrix	*w*_*n*_	sensor noise matrix

The Proportional Integral Derivative (PID) control, discussed in [10, 11] as a fractional controller, and further elaborated in [12] for the coupled system’s degree of freedom, is one approach. Nonlinear control combined with estimation control for addressing highly nonlinear systems is detailed in [13]. Swarm optimization is employed to optimize the decoupled system, building upon the PID control strategy presented in [14]. Classical control strategies, defined with a focus on the efficient output response of UAVs, are explained in [15, 16]. The integration of nonlinear control with classical control techniques is demonstrated in [17]. To eliminate coupling effects, decoupling techniques, whether static or dynamic, are utilized.

In [18, 19], adaptation laws based Model Predictive Control (MPC) strategy with neural networks are introduced to overcome the parametric variations. Furthermore, an adaptive MPC approach via a learning-based strategy is explored in [20, 21]. An alternative control strategy for uncertainties is Multiple Model Second-Level Adaptation (MALSA), as described in [22, 23]. In addition, an intelligent control strategy, tailored to the prototype’s nonlinear behavior detailed in [24], is implemented in a discrete-time framework. This strategy comes in two variants, sliding mode control and integral SMC, both designed to address the response of the system’s states following the use of a decoupler, as presented in [16, 25]. An uncertain quadrotor UAV should be able to track a trajectory with model uncertainties, external disturbances, actuator faults, and input delay. To achieve this, research in [26] proposed a control scheme based on disturbance observer and sliding mode manifold. A neural network-based control scheme, specifically a radial basis function neural network (RBFNN) with robust approximation capabilities, is designed to compensate for uncertainties, disturbances, and actuator faults for fault tolerance [27]. Another research addressing uncertainties, time delays, external disturbances and non-linearities is reported in [28], which proposed a control approach involving an equivalent input disturbance (EID) estimator. The estimator is used for external disturbances and non-linearities.

The authors in [29–31] presented controller design considering uncertain nonlinear systems. The presented control strategy in both of the research works employed Lyapunov functions and Linear Matrix Inequality (LMI) conditions to ensure stability and optimal performance despite uncertainties and disturbances. Another important strategy for designing robust *H*_∞_ controllers especially for Linear Time Invariant (LTI) systems is based on the Riccati equation. This approach is often used in conjunction with the LMI approach [32, 33]. Most of the aforementioned control strategies including [29–33] suffer from some drawbacks. LMI approach and Riccati equation for *H*_∞_ robust control design can be conservative, leading to more robust controllers but less optimal in terms of performance due to higher-order nonlinear and coupled dynamics of the system [31, 33]. Also, this strategy requires accurate models of uncertainty including bounds on uncertain parameters. However, in real-world systems, obtaining precise models of uncertainty can be challenging. If the uncertainty model is not accurately represented, the designed control strategy may not perform as expected. In contrast to [29–33], the controller proposed in the present research has the ability to reduce conservatism, and owing to its flexible nature, the controller can be extended to handle complex nonlinear systems. Moreover, the proposed method also allows for the inclusion of constraints in the control design process. Constraints can be imposed on control inputs, states, or outputs to ensure that the system operates within desired bounds or satisfies specific requirements. Loop Shaping Control (LSC), while useful, struggles with these complexities, especially in system modeling and stability analysis. Additionally, the design process for these systems is computationally demanding, leading to longer development times and implementation difficulties. These UAVs are also highly sensitive to uncertainties, including modeling errors and external disturbances, which can compromise controller performance and stability. Similarly, LSC limits its ability to address other control aspects, such as robustness to disturbances or transient responses. The key novelty of our work lies in proposing and developing a flexible mixed-optimization approach with *H*_∞_ control for a coupled UAV system, which utilizes the method of inequality (MOI). This novel framework allows us to address the complex coupling dynamics of the UAV system and design an effective control strategy that balances diverse optimization objectives, such as stability, performance, robustness, and reduced conservatism. Moreover, in contrast to most of the reported works on nonlinear control and optimization of UAVs including [30–33] are limited to simulation, the present work reports experimental results obtained from a helicopter prototype, thus evidencing the real-world applicability of the proposed optimization approach.

In our previous studies [25, 34], we have considered the nonlinear characteristics and the coupling effect between the rotors as perturbations. To address the complexities associated with inverting the motion equations and reduce model complexity, we have employed a controller design based on Non-linear Dynamic Inversion (NDI). The stability of the system is ensured through calculations of the controllability and observability matrices. These calculations yield full rank matrices of sizes 6*10 and 10*6, confirming the controllability and observability of the system. Furthermore, we apply a decoupling technique to transform the system into two subsystems: the Vertical Plane System (VPS) and the Horizontal Plane System (HPS). Quantitative measurements play a vital role in the experimental response of control systems, particularly in the case of UAVs. These measurements are given significant attention as they offer valuable insights into various aspects of system behavior, stability analysis, response speed, stability, accuracy, and system identification. These properties are critical for the development of effective control strategies and the optimization of performance in real-world applications. Higher-order UAV systems present significant challenges in control design due to their complex dynamics and states. This research pioneers a groundbreaking mixed optimization technique in *H*_∞_ model-based control for MIMO systems, marked by several key contributions:

1. **Innovative flexible-nature design strategy:** We introduce an innovative approach characterized by its flexibility. This strategy utilizes weighted functions as tuning parameters, integral to the optimization process. These weighted functions, when combined with *H*_∞_ model-based control, create a robust and sophisticated optimization framework.
2. **Unified mixed optimization framework:** The integration of weighted functions with *H*_∞_ control forms a unique mixed optimization model. This integration, leveraging the Method of Inequality (MOI), enhances the optimization process, yielding comprehensive outcomes in system performance and stability.
3. **Adaptability and robust performance:** The proposed method allows for the adjustment of design parameters, enhancing the algorithm’s maturity and robustness. This adaptability proves crucial in managing internal and external perturbations, including the unstructured dynamics prevalent in MIMO systems, thereby ensuring stable and robust performance in various complex scenarios.
4. **Real-time application in challenging conditions:** Demonstrated through real-time applications under challenging conditions, including noise, parametric variations, disturbance torque, and wind disturbances, the method validates its robust optimization capabilities. It shows improved system responses compared to current research findings, highlighting its effectiveness in practical scenarios.

The remainder sections are structured as follows. The modeling of the system is presented in mathematical modeling. The Loop Shaping Control (LSC), mixed-sensitivity control introduction with its limitations and mixed optimization design procedure are elaborated in the control design. The simulation and experimental response to validate the robustness is verified in the simulation results. The conclusion offers an in-depth comparison, including a comprehensive table displaying the simulation results.

## 2 Mathematical model

**Fig 1:**
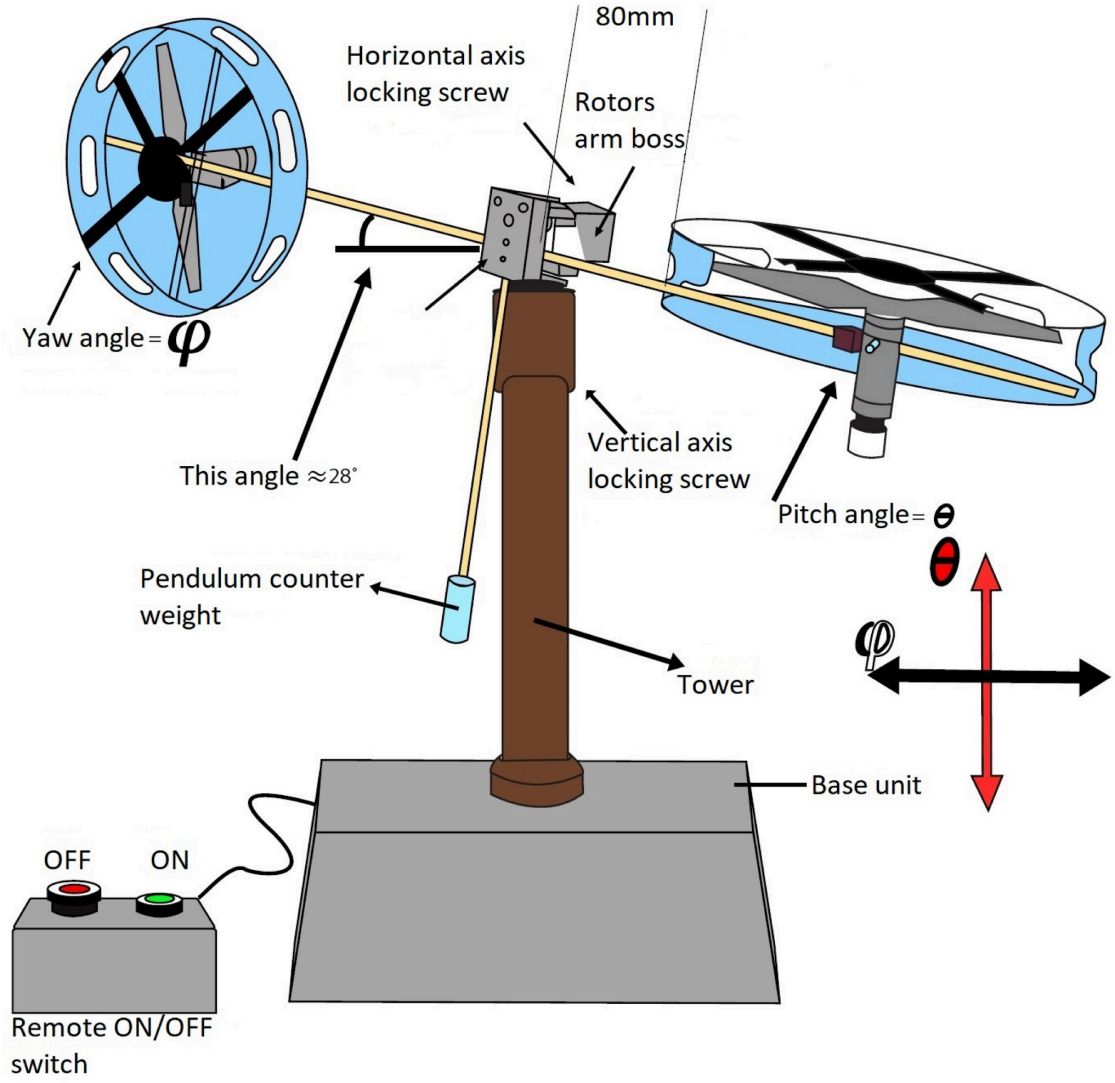
Basic schematic sketch of TRMS [35].

**Fig 2:**
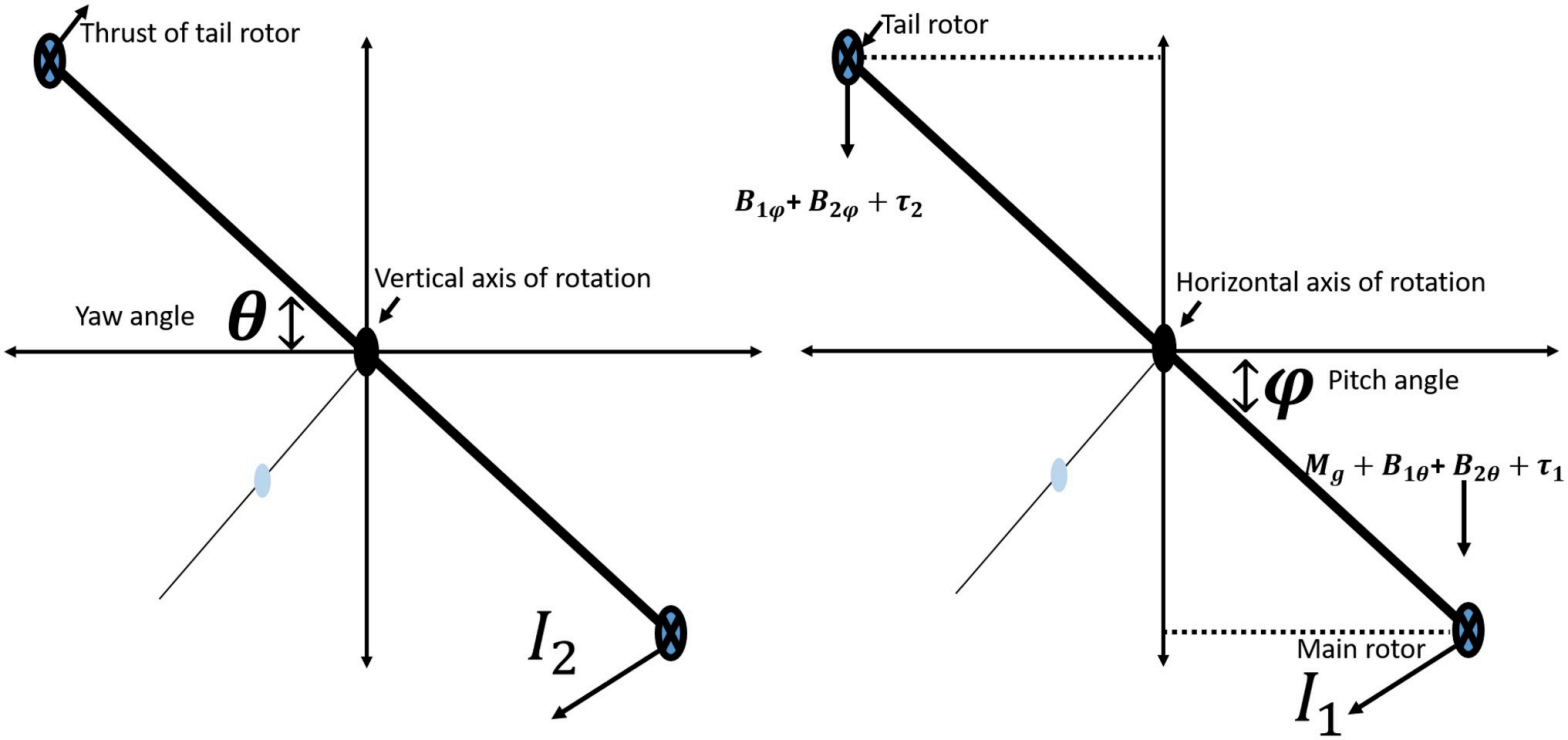
Phenomenological model with rotors dimension.

A nonlinear system’s modeling can be established by making certain necessary, though judicious, assumptions, as outlined in [35]. We depict a system with limited movement, along with the key parameters, as illustrated in Fig 1. Twin rotor MIMO system typically consists of two independently controlled rotors, often placed in a parallel configuration represented in Fig 2. Each rotor can produce thrust and torque, allowing for control of the system’s position and orientation in space. The mathematical modeling of the UAV is given below under certain assumptions for simplification in understanding the system. The mechanical part of the UAV is elaborated via mathematical expression in Eq (1) as a momentum of pitch angle.

$$\begin{array}{c} \;I_{1}\ddot{\theta }=M_{1}-M_{FG}-M_{B\theta }-M_{G}\; \end{array}$$ (1)

$$\begin{array}{c} \;M_{1}=a_{1}\tau_{1}^{2}+b_{1}\tau_{1}\; \end{array}$$ (2)

$$\begin{array}{c} \;M_{FG}=M_{g}\;\text{sin}\;\theta \; \end{array}$$ (3)

$$\begin{array}{c} \;M_{B\theta }=B_{1\theta }\dot{\theta }+B_{2}\theta \;\text{sin}\left(2\theta \right){\dot{\varphi }}^{2}\; \end{array}$$ (4)

$$\begin{array}{c} \;M_{G}=K_{gy}M_{1}\dot{\varphi }\;\text{cos}\left(\theta \right)\; \end{array}$$ (5)

where *a*_1_ and *b*_1_ are constants. This equation is based on the law of conservation of angular momentum. The Eq (6) is a mathematical expression of the tail rotor momentum.

$$\begin{array}{c} \;I_{2}\ddot{\varphi }=M_{2}-M_{B\varphi }-M_{R}\;\; \end{array}$$ (6)

The momentum equations in the vertical plane of motion are written as,

$$\begin{array}{c} \;M_{2}=a_{2}\tau_{2}^{2}+b_{2}\tau_{2}\;\; \end{array}$$ (7)

$$\begin{array}{c} \;M_{B\varphi }=B_{1\varphi }\dot{\varphi }\; \end{array}$$ (8)

$$\begin{array}{c} \;M_{R}=K_{c}\frac{T_{o}s+1}{T_{p}s+1}M_{1}\;\; \end{array}$$ (9)

The Runge-Kutta method is a powerful numerical integration technique for approximating solutions to ordinary differential equations (ODEs). It offers several properties that make it widely used and suitable for various applications. The Runge-Kutta method’s combination of accuracy, stability, simplicity, and versatility makes it a fundamental and widely used numerical integration technique in scientific and engineering disciplines. The objective is to design a model accurately by Runge-Kutta. The prototype featuring both rotors, including the main and tail rotor, can be mathematically described by the following state equations:

### 2.1 Equation for the main rotor (*θ*)

$$\begin{array}{c} \frac{d\dot{\theta }}{dt}=\frac{a_{1}}{I_{1}}\tau_{1}^{2}+\frac{b_{1}}{I_{1}}\tau_{1}-\frac{M_{g}}{I_{1}}\;\text{sin}\left(\theta \right)+\frac{0.0326}{2I_{1}}\;\text{sin}\left(2\theta \right){\dot{\varphi }}^{2}-\frac{B_{1\theta }}{I_{1}}\theta -\frac{k_{g_{y}}B_{2\theta }}{I_{1}}\;\text{cos}\left(\theta \right)\dot{\varphi }(a_{1}\tau_{1^{2}}+b_{1}\tau_{1}) \end{array}$$ (10)

### 2.2 Equation for the tail rotor (*φ*)

$$\begin{array}{c} \frac{d\;\dot{\varphi }}{dt}=\frac{a_{2}}{I_{2}}\tau_{2}^{2}+\frac{b_{2}}{I_{2}}\tau_{2}-\frac{B_{1\varphi }}{I_{2}}\dot{\varphi }-\frac{k_{c}B_{2\varphi }}{I_{2}}1.75(a_{1}\tau_{1}^{2}+b_{1}\tau_{1}) \end{array}$$ (11)

The above state-space equations provide a comprehensive representation of the dynamics and interactions between the main and tail rotors in the system. They are instrumental in analyzing and controlling the system’s behavior to ensure stability and achieve desired performance. The mathematical equation for the main rotor can be expressed as follows:

$$\begin{array}{c} \;{\dot{\tau }}_{1}=\frac{T_{10}}{T_{11}}\tau_{1}+\frac{k_{1}}{T_{11}}u_{1} \end{array}$$ (12)

Eq (12) captures the dynamics of the main rotor, including the effects of stiffness, and external control inputs. It describes how the pitch angle of the main rotor changes over time in response to these factors. By manipulating the control input, it is possible to regulate the main rotor’s behavior and achieve the desired motion along the vertical axis (vertical plane). Similarly. the mathematical equation for the tail rotor can be expressed as follows:

$$\begin{array}{c} \;{\dot{\tau }}_{2}=\frac{T_{20}}{T_{21}}\tau_{2}+\frac{k_{2}}{T_{21}}u_{2} \end{array}$$ (13)

Eq (13) describes the dynamics of the tail rotor, taking into account factors such as damping, stiffness, and external control inputs. It governs how the yaw angle of the tail rotor evolves over time, influencing the motion along the horizontal axis (horizontal plane). The state Eqs (10)–(13) have been transformed into linear state equations, wherein no term exceeds a degree of one. Linearization is the method used to derive these linear equations, albeit with certain necessary simplifying assumptions. The model can be characterized as follows:

$$\begin{array}{c} \;\dot{x}(t)=Ax(t)+Bu(t) \end{array}$$ (14)

$$\begin{array}{c} \;y(t)=Cx(t) \end{array}$$ (15)

Here, *x* represents the states, which are real numbers (x∈R), while *u* denotes the signal as input and *y* signifies the output, all within the realm of real numbers (u,y∈R). The state equations of TRMS are provided below:

$$\begin{array}{c} x={\left[\;\;\theta \;\;\;\;\;\;\;\;\;\dot{\theta }\;\;\;\;\;\;\;\;\varphi \;\;\;\;\;\;\;\;\dot{\varphi }\;\;\right]}^{T} \end{array}$$ (16)

$$\begin{array}{c} \;y={\left[\;\;\theta \;\;\;\;\;\varphi \;\;\right]}^{T} \end{array}$$ (17)

In this context, *θ* signifies the pitch motion, *ϕ* denotes the yaw motion, *τ*_1_ corresponds to the momentum for the pitch position rotor, and *τ*_2_ stands for the momentum of the tail rotor. The application of NDI involves streamlining the system through straightforward mathematical calculations. The states of the system, when linearized at the origin, can be expressed as *x*(*t*) = *x*(0).

$$\begin{array}{c} J=\frac{\delta f(x)}{\delta x}{|}_{x=0} \end{array}$$ (18)

J=[∂f1∂x1⋯∂f1∂x6⋮⋱⋮∂f6∂x1⋯∂f6∂x6] (19)

Several mathematical operations have been applied to derive the simplified matrices at the origin, represented as (0, 0, 0), and these are presented below:

$$\begin{array}{c} A=[\begin{array}{cccccc} 0 & 1 & 0 & 0 & 0 & 0\\ -\frac{M_{g}}{I_{1}} & -\frac{B_{1\theta }}{I_{1}} & 0 & 0 & \frac{b_{1}}{I_{1}} & 0\\ 0 & 0 & 0 & 1 & 0 & 0\\ 0 & 0 & 0 & -\frac{B_{1\varphi }}{I_{2}} & -\frac{k_{c}}{I_{2}}1.75 & \frac{b_{2}}{I_{2}}\\ 0 & 0 & 0 & 0 & -\frac{T_{10}}{T_{11}} & 0\\ 0 & 0 & 0 & 0 & 0 & -\frac{T_{20}}{T_{21}} \end{array}] \end{array}$$ (20)

$$\begin{array}{c} C=[\begin{array}{cccccc} 1 & 0 & 0 & 0 & 0 & 0\\ 0 & 0 & 1 & 0 & 0 & 0 \end{array}]B=[\begin{array}{cc} 0 & 0\\ 0 & 0\\ 0 & 0\\ 0 & 0\\ \frac{k_{1}}{T_{11}} & 0\\ 0 & \frac{k_{2}}{T_{21}} \end{array}] \end{array}$$ (21)

**Fig 3:**
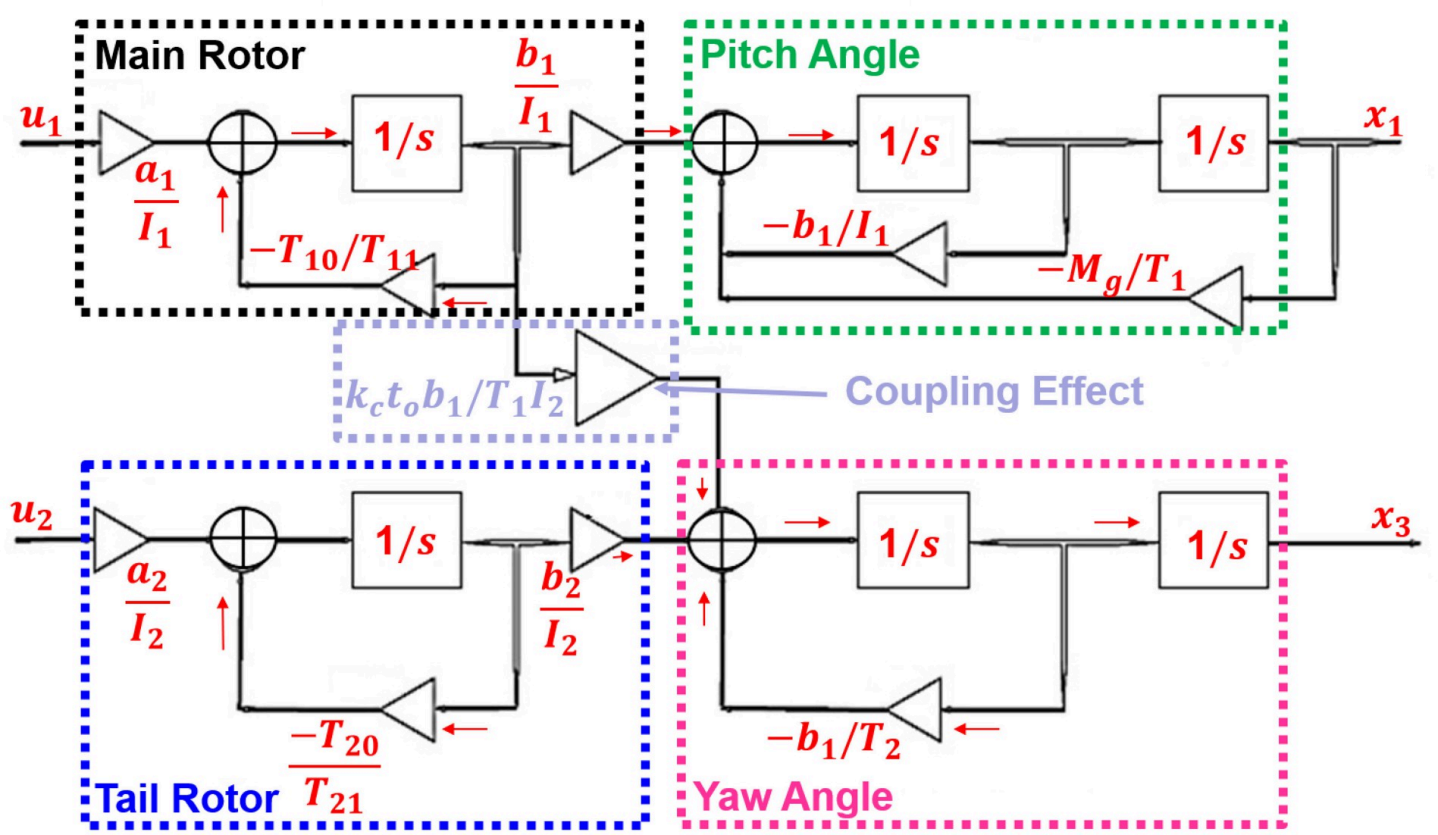
Block diagram of the MIMO system.

Table 2 furnishes the system’s parametric values, along with accompanying descriptions. Fig 3 illustrates the block diagram of the UAV, capturing the disturbance torque known as the coupling effect. Within this diagram, there are two states referred to as the pitch position and yaw position and the disturbance torque between the rotors.

**Table 2:** Parameters of UAV [35].

Symbol	Description	Value	Unit
*I*_1_	main rotor inertia	6.8 × 10^−2^	*kgm* ^2^
*I*_2_	tail rotor inertia	2 × 10^−2^	*kgm* ^2^
*a*_1_	main motor constant	0.0135	-
*b*_1_	main motor constant	0.0924	-
*a*_2_	tail motor constant	0.02	-
*b*_2_	tail motor constant	0.9	-
*M*_*g*_	gravitational momentum	0.32	*Nm*
*B*_1*θ*_	frictional parameter	6 × 10^−3^	*Nms*^2^/*rad*^2^
*B*_2*θ*_	frictional parameter	1 × 10^−3^	*Nms*^2^/*rad*^2^
*B*_1*φ*_	frictional parameter	1 × 10^−1^	*Nms*/*rad*
*B*_2*φ*_	frictional parameter	1 × 10^−2^	*Nms*^2^/*rad*
*k*_*gy*_	gyroscopic parameter	0.05	*rad*/*s*
*k*_1_	gain of main motor	1.1	-
*k*_2_	gain of tail motor	0.8	-
*T*_11_, *T*_10_, *T*_21_, *T*_20_	constants of motor	1	-
*k*_*c*_	coupling reaction for gain	2	-

### 2.3 Stability analysis of TRMS (UAV)

The system under examination is subject to a mathematical assessment known as controllability and observability. This necessitates that all dynamic states of the model be controllable and is determined by the system matrices *A* and *B* called controllability. In Eq (22), we encounter a linear system matrix referred to as controllable form, indicating the ability to find an input *u*(*t*) that can drive the state *x*(*t*_*o*_) to the origin within a finite time. If there exists an input that satisfies the condition *x*(*t*) = 0 for all initial times and states, it confirms the system’s controllability, as discussed in [36]. The controllability matrix is elucidated as follows:

- (A, B) is controllable,
- The controllability of the system:
  $$\begin{array}{c} C_{c}=\left[B\;AB\;A^{2}B\;A^{3}B\;A^{4}B\;...\;{A^{\alpha -}}^{1}B\right] \end{array}$$ (22)

Here, *α* represents a positive integer that varies depending on the order of the system matrix *A*,” with Eq (22) having a matrix order of 6. A system is deemed controllable when its determinant is non-zero. The matrix in question exhibits full rank, affirming the system’s controllability. This full rank property further solidifies the system’s controllability. The resulting controllability matrix is as follows:

$$C_{c}=[\begin{array}{cccccccccc} 0 & 0 & 0 & 0 & 0.0014 & 0 & -0.0014 & 0 & -0.0052 & 0\\ 0 & 0 & 0.0014 & 0 & -0.0014 & 0 & -0.0052 & 0 & 0.0058 & 0\\ 0 & 0 & 0 & 0 & 0.0016 & 0.0036 & -0.0096 & -0.0216 & 0.0491 & 0.1116\\ 0 & 0 & -0.0016 & 0.0036 & 0.0096 & 0.0216 & 0.0491 & 0.1116 & 0.2468 & 0.5616\\ 1 & 0 & 0.0009 & 0 & 0.0008 & 0 & 0.0008 & 0 & 0.0007 & 0\\ 0 & 0.8 & 0 & 0.0008 & 0 & 0.0008 & 0 & 0.0008 & 0 & 0.0008 \end{array}]$$ (23)

The evident illustration of the system’s full-rank characteristic is evident in the matrix displayed above, exemplifying the attributes of a full-rank matrix. To affirm this full-rank property, one may calculate the determinant of the system matrix, which should not yield a result of zero. Additionally, when all system states converge to the origin, it serves as confirmation of the system’s observability. Observability, which acts as the dual counterpart to controllability, can be ascertained in the following manner:

- (A, C) is observable,
- The observability matrix can be found here,
  $$\begin{array}{c} O_{O}={\left[C\;CA\;CA^{2}\;CA^{3}\;CA^{4}\ldots \ldots C{A^{\alpha -}}^{1}\right]}^{T} \end{array}$$ (24)

For the system to be deemed controllable, it’s crucial that its determinant is not equal to zero. As the matrix demonstrates the full rank property, it confirms the observability of the system. The computed observability matrix is presented below, and its full rank property underscores the system’s observability. Stability analysis lays a robust foundation for designing an appropriate controller. In the subsequent section, we delve into the controller constraints as per the system dynamics.

$$\begin{array}{c} O_{O}=[\begin{array}{cccccc} 1 & 0 & 0 & 0 & 0 & 0\\ 0 & 0 & 1 & 0 & 0 & 0\\ 0 & 1 & 0 & 0 & 0 & 0\\ 0 & 0 & 0 & 1 & 0 & 0\\ -4.7059 & -0.0882 & 0 & 1.3588 & 0 & 0\\ 0 & 0 & 0 & -5 & 1.6170 & 4.5\\ 0.4151 & -4.6534 & 0 & 0 & -1.3543 & 0\\ 0 & 0 & 0 & 25 & -9.5532 & -27\\ 22.1089 & 0.8294 & 0 & 0 & -8.6574 & 0\\ 0 & 0 & 0 & -125 & 49.1258 & 139\\ -3.9032 & 22.0356 & 0 & 0 & 5.8103 & 0\\ 0 & 0 & 0 & 663 & -246 & -702 \end{array}] \end{array}$$ (25)

**Fig 4:**
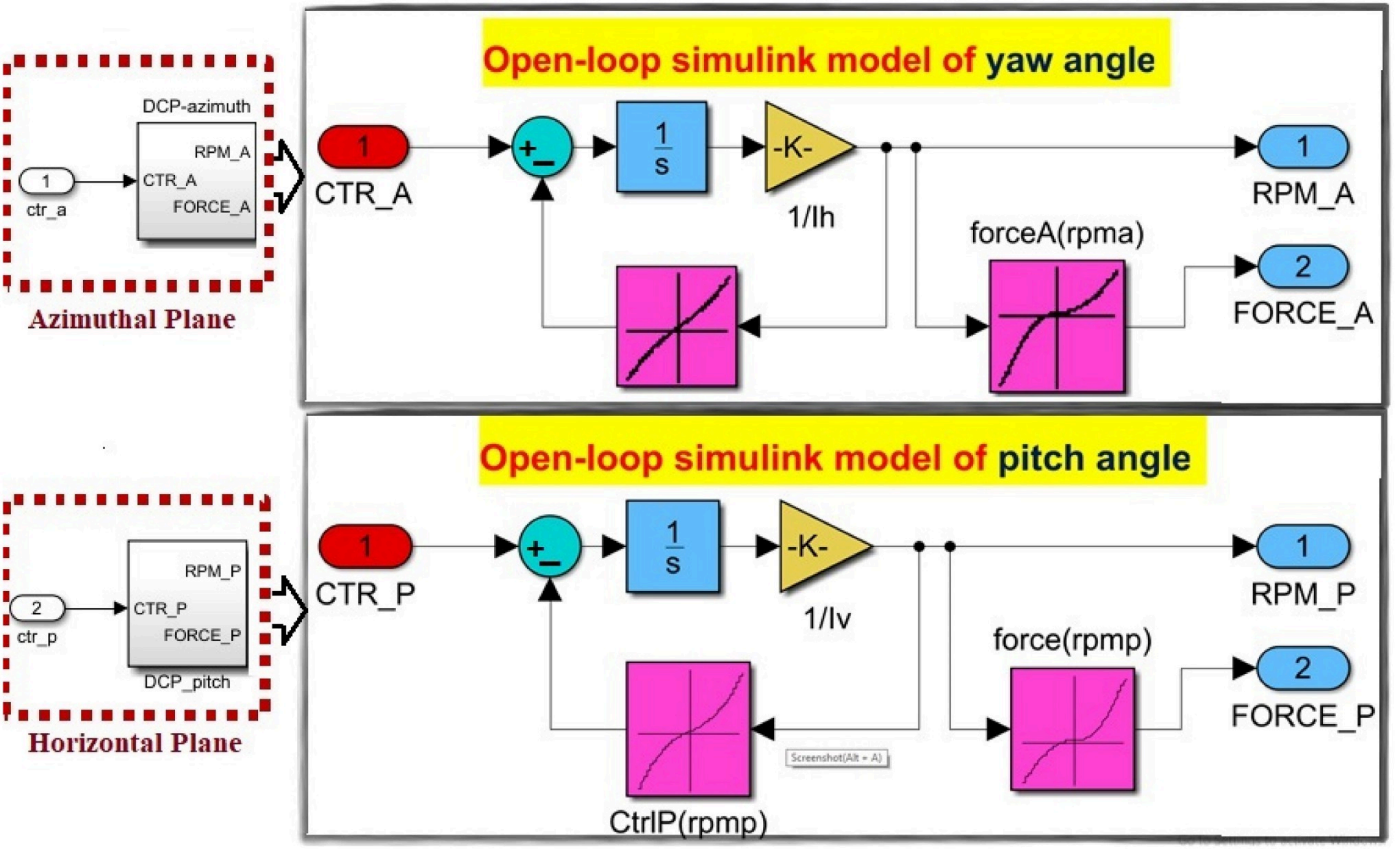
Block diagram of the SISO subsystems.

The cross-coupling effect with all other unwanted perturbations makes the TRMS behavior very complex. The horizontal plane angle can be fixed by posing the value of *u*_2_ = 0. Decoupling makes the system into subsystems such as VPS and HPS represented in Fig 4. The transfer function of both subsystems is given below:

$$\begin{array}{c} G_{\theta }(s)=\frac{111.2}{0.390s^{3}+0.3835s^{2}+1.454s+1} \end{array}$$ (26)

$$\begin{array}{c} G_{\phi }(s)=\frac{111.2}{5.64s^{2}+3.97s+1} \end{array}$$ (27)

where *G* = [*G*_*θ*_ *G*_*ϕ*_]^*T*^ and subsystems are obtained by putting second control input equal to zero. During the inversion process, NDI may encounter singularity issues that can affect its performance. This singularity can alter the rank of the system matrix, leading to discontinuous behavior and potentially causing unbounded elements in the matrix. To mitigate these drawbacks, a solution is proposed in [37], which involves augmenting a scaling factor, as elaborated below:

$$\begin{array}{c} \dot{v}(t)=-v(t)+\frac{\gamma }{e_{z}{\left(t\right)}^{2}},\;v(0)>0 \end{array}$$ (28)

where *γ* is constant and *e*_*z*_(*t*) represents the tracking of pitch angle and yaw angle. A negative sign represents the convergence towards origin and asymptotic stability confined with tracking control of the angles in the above expression.

## 3 Control design

Linear control design is important for nonlinear coupled systems primarily because it provides a means to simplify and manage the complexity of such systems, enabling effective control. Designing controllers for linearized models is often more straightforward and allows for the application of well-understood control strategies.

### 3.1 Loop Shaping Control (LSC)

**Fig 5:**
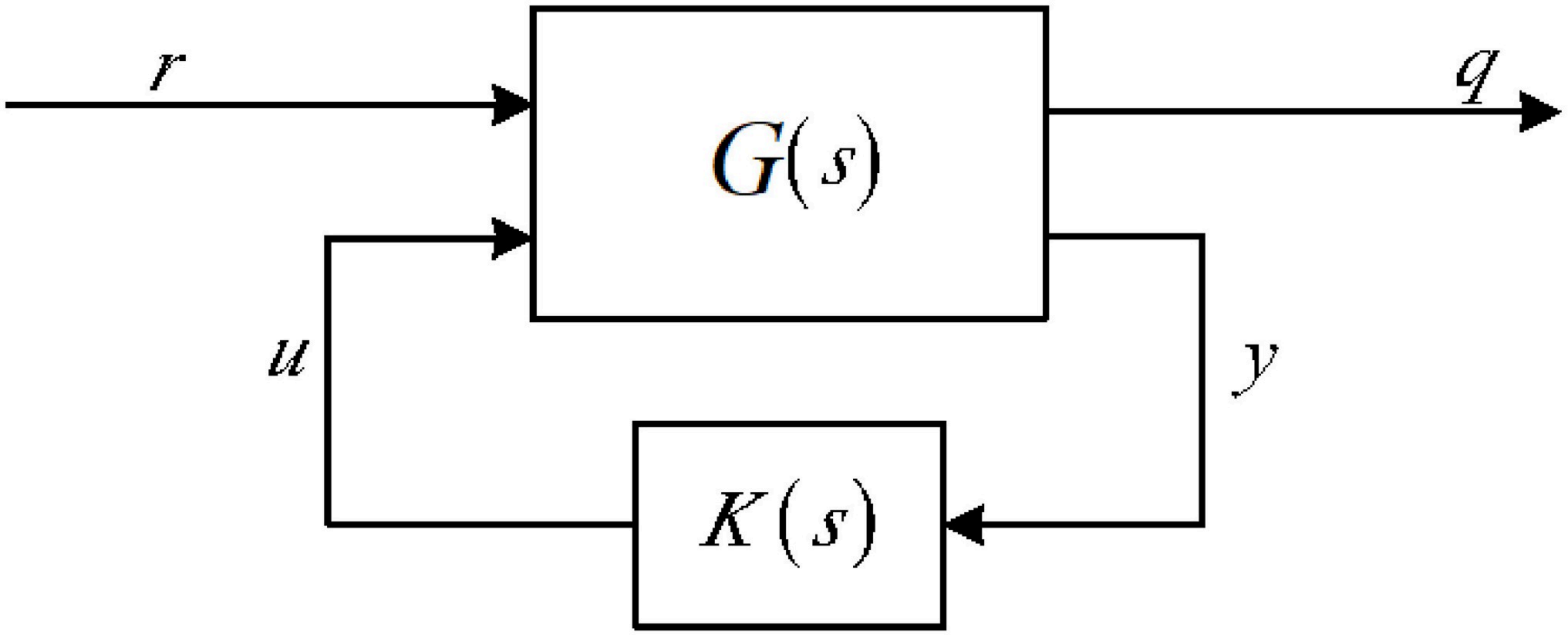
Standard LSC configuration.

The attainment of nominal performance and robust stabilization hinges on the particular cost function of the LSC problem, as detailed in [38] and illustrated as a sub-optimization in Fig 5.

In the context of highly nonlinear closed-loop systems, the utilization of a controller gain denoted as ‘K’ becomes crucial in maintaining a seamless output by managing the control input ‘U’. On the other hand, ‘G’ signifies a generic nonlinear system that reacts to a reference input ‘r’. To create a robust controller, different formulas are at one’s disposal, each tailored to meet specific design criteria, ST and SKS weighted method. Optimal result can be obtained by SKS, In this context, the variable *S* corresponds to sensitivity, and the output gain of the controller is denoted as *K*. This function serves the purpose of attenuating output disturbances, ensuring accurate tracking of desired responses, and enhancing the model’s robust stability against additive perturbations. Robust performance in the presence of multiplicative model uncertainty can be attained through the following function ST, In this context, *T* represents the complementary sensitivity function. Both methods for designing robust controllers are valuable and applicable, but they have limitations arising from the representation of model uncertainty. These limitations are linked to the number of poles located in the right half of the complex plane, as discussed in [39]. It’s important to note that there is a possibility of pole-zero cancellation when working with a nominal model and during the controller design process. Alternatively, there’s another approach to introducing perturbations, which leads to the presentation of robust stabilization design methods. To circumvent pole-zero cancellations in the right half-plane, the LSC method is introduced. The LSC relaxes the constraint on the number of poles in the right half-plane within both the nominal model and the controller.

The coprime factorization can be elaborated in matrices, denoted as $\left(\tilde{M},\tilde{N}\right)\in H_{\infty }^{+}$, elucidates across the state space for a prototype model *G*. This representation holds if and only if certain conditions are met:

-
$\tilde{M}=0$ , $det(\tilde{M})\neq 0$
- Represents the model $\;G=\;{\tilde{M}}^{-1}\tilde{N}$
- The value of $\left(\tilde{V},\tilde{N}\right)\in H_{\infty }^{+}$, $\tilde{M}\tilde{V}+\tilde{N}\tilde{U}=I$

The plant is normalized for the left coprime factorization, if and only if,

$$\begin{array}{c} \tilde{M}\tilde{M}+\tilde{N}\tilde{N}=I \end{array}$$

The state equations for normalized factorization are obtained through equation as,

$$\begin{array}{c} \left(A-BD^{t}R^{\left(-1\right)}C\right)Z+Z\left(A-BD^{t}R^{\left(-1\right)}C\right)-ZC^{T}R^{\left(-1\right)}CZ+B\left(I-D^{T}R^{\left(-1\right)}D\right)B^{T}=0 \end{array}$$

where *R* = *I* + *DD*^*T*^ and *D* ≥ 0 is unique solution for stabilization.

$$\begin{array}{c} \left[\tilde{N}\;\;\tilde{M}]=\;[\frac{A+HC\;|B+HD\;\;\;H}{N^{-1/2}C|R^{-1/2}DR^{-1/2}}\right] \end{array}$$

The desired outcome for the normalization of the factorization of $G={\tilde{M}}^{-1}\tilde{N}$ can be obtained. Likewise, the normalization of the factorization can be computed as follows:

$$\begin{array}{c} {\left(A-BS^{\left(-1\right)}D^{T}C\right)}^{T}X+X\left(A-BS^{\left(-1\right)}D^{T}C\right)-XBS^{\left(-1\right)}B^{T}X+C^{T}\left(I-DS^{\left(-1\right)}D^{T}D\right)C=0 \end{array}$$

here *s* = *i* + *D*^*T*^*D* and *X* ≥ 0. The perturbation of the plant shown as:

$$\begin{array}{c} G_{\Delta ;}=(\tilde{M}+G_{\Delta }){\left(\tilde{M}+\Delta_{\tilde{M}}\right)}^{-1}{\left(\tilde{N}+\Delta_{\tilde{N}}\right)}^{-1} \end{array}$$

Here, $(\Delta_{\tilde{M}},\Delta_{\tilde{N}}$) denotes the unstructured perturbation. We can attain the Hankel computational approximation of the controller K through the following process:

$$\begin{array}{c} K=\;[\frac{A+BF+\gamma^{2}(L^{T}-1)ZC^{T}(C+DF)\;\;\;|\;\;\;\;\gamma^{2}(L^{T}-1)ZC^{T}}{B^{T}X\;\;\;\;\;\;\;\;|\;\;\;\;\;\;-D^{T}}] \end{array}$$

In this context, let’s define *F* = −*S*^−1^(*D*^*T*^*C* + *B*^*T*^*X*) and *L* = (1 − *γ*^2^)*I* + *XZ*. Under certain conditions, it may exhibit singular behavior, making it impractical for implementation. To address this issue, the controller gain can be computed as follows:

$$\begin{array}{c} K=\;[\frac{-L^{T}s+L^{T}(A+BF)+\gamma^{2}ZC^{T}(C+DF)\;\;\;\;|\;\;\;\;\;\;\gamma^{2}ZC^{T}}{B^{T}X\;\;\;\;\;\;\;\;|\;\;\;\;\;\;-D^{T}}] \end{array}$$

The primary objective of the optimization task is to eliminate both structured and unstructured perturbations while achieving a smooth output without overshooting. By optimizing the system’s gain, we can effectively address singularity issues and minimize the cost function. The intended frequency response must adhere to particular requirements, encompassing robust stability, robust performance, and specified conditions for crossover frequency and roll-off. Implementing the LSC approach yields square input responses, which in turn aid in achieving convergence for both pitch and yaw angles towards the desired trajectory. This convergence is visually depicted in Figs 16 and 17, respectively. Furthermore, to gain a deeper understanding of the higher-order UAV’s behavior, we delve into the concept of mixed sensitivity in the subsequent subsection.

### 3.2 Mixed sensitivity design strategy

**Fig 6:**
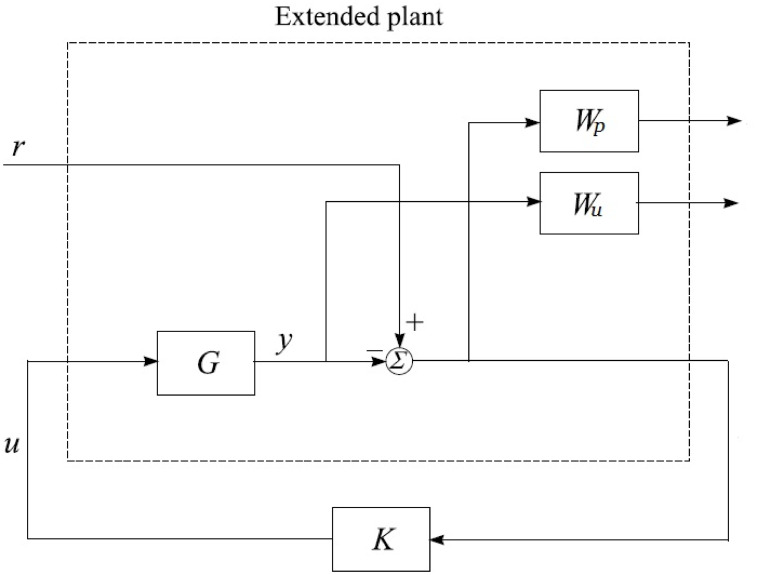
Mixed-sensitivity approach.

A system is considered robust when it can maintain optimal performance and stability with considered disturbances, as outlined in [40]. The concept of mixed sensitivity control involves minimizing the cost function by focusing on sensitivity attributes at low frequencies, as discussed in [41]. Robust controllers are specifically designed for closed-loop systems, and this technique, which has evolved over the past two decades, has consistently delivered effective results. To address various nominal and robust stability concerns and minimize issues related to the *H*_∞_ norm, the *H*_∞_ mixed-sensitivity approach is employed. This approach not only resolves nominal performance challenges, as depicted in Fig 6, but also tackles robust optimization problems, as mentioned in [39]. A robust strategy aims to achieve a satisfactory, smooth output. The conventional procedure for designing a controller to overcome the specific problem involves the use of a transfer function for a general plant *G*(*s*) with a representation of the closed-loop transfer function.

$$\begin{array}{c} G(s)=[\begin{array}{cc} g_{11}(s) & g_{12}(s)\\ g_{21}(s) & g_{22}(s) \end{array}] \end{array}$$

$$\begin{array}{c} z=F_{l}\left(P,K\right)w \end{array}$$

$$\begin{array}{c} z=[g_{11}+g_{12}K{\left(I-g_{22}\right)}^{-1}g_{21}=\;F_{l}(P,K)w \end{array}$$

Here, *F*_*l*_(*P*, *K*)*w* confirms the utilization of a transform involving *P* and *K*. The objective for the controller under consideration is as follows:

$$\begin{array}{c} K_{stabilizing}^{min}F_{l}(G,K)w_{\infty } \end{array}$$

The optimization possesses the capability to optimize the norm of infinity, which should result in a smooth controller gain, denoted as K. The primary responsibility of this gain is to reduce:

$$\begin{array}{c} F_{l}(G,K)w_{\infty }=max_{w}\bar{\sigma }(F_{l}(G,K)(jw)) \end{array}$$

The challenge of sub-optimality and interconnectivity within the plant can be framed as:

$$\begin{array}{c} F_{l}(G,K)w_{\infty }<\gamma \end{array}$$

$$\begin{array}{c} G=[\begin{array}{cc} I & -MW_{1}\\ 0 & I\\ I & -MW_{2} \end{array}] \end{array}$$

Generally, the weighting functions *W*_*P*_, *W*_*u*_ are weighted (tuning variables) matrices with appropriate dimensions. However, LSC and *H*_∞_ mixed sensitivity are not without their drawbacks, especially when dealing with higher-order UAV systems. Higher-order UAV systems present significant challenges in control design due to their complex dynamics and states. Loop Shaping Control, while useful, struggles with these complexities, especially in system modeling and stability analysis. Additionally, the design process for these systems is computationally demanding, leading to longer development times and implementation difficulties. These UAVs are also highly sensitive to uncertainties, including modeling errors and external disturbances, which can compromise controller performance and stability. LSC’s focus on frequency domain shaping limits its ability to address other control aspects, such as robustness to disturbances or transient responses. Moreover, these systems often lack adaptability to changes over time, impacting long-term performance. Achieving a balance between performance specifications and stability is another critical challenge, where prioritizing one can negatively impact the other. The controllers also show limitations in robust performance validation and real-time response effectiveness. Even the mixed sensitivity approach using the *H*_∞_ technique does not guarantee robust stability and performance, particularly in highly coupled systems, often nearing stability thresholds of about 60%.

The model-based *H*_∞_ control is enhanced with mixed optimization control, where weighting parameters are treated as design parameters. This approach offers significant advantages in terms of robustness. The controller’s objective is to meet the criteria for the real-time performance of the system, which will be discussed in the following subsection.

### 3.3 Mixed optimization with robust performance validation

The selection of the gain is closely intertwined with the choice of weighting functions, which, in turn, are determined by evaluating the open-loop response of the weighted plant. In essence, the weights represented by *W*_*p*_ and *W*_*u*_ are treated as design parameters. This approach implies that the design challenge can be reconfigured so that the parameters of the weighting functions serve as design variables aimed at satisfying a set of closed-loop performance constraints, as elaborated in [37]. The designer’s responsibility is not to determine the controller’s order but rather to determine the order of the weighting functions. By utilizing low-order weighting functions, it becomes possible to synthesize high-order controllers, often leading to significantly enhanced performance or robustness compared to the utilization of simple low-order controllers. The design problem can now be articulated as follows: Start by defining the plant *G* and specifying its characteristics. (i) Define the values of *e*_*y*_ and *e*_*z*_ to establish the desired closed-loop performance. (ii) Specify the form and initial order of the weighting functions *W*_*p*_ and *W*_*u*_. Ensure that bounds are set to maintain stability and minimum phase properties for *W*_*p*_ and *W*_*u*_, preventing unwanted pole/zero cancellations. It’s advisable to start with a small initial order for the weighting functions. (iii) Set the initial values of *W*_*p*_ based on the open-loop frequency response of the plant. (iv) Employ appropriate algorithms to search for a combination of (*W*_*p*_, *W*_*u*_) that satisfies the specified inequalities. If a solution is found, the design is considered satisfactory. If not, consider options such as increasing the order of the weighting functions, relaxing some of the desired bounds, or attempting the design process again. (v) Once satisfactory weighting functions *W*_*p*_ and *W*_*u*_ are identified, you will have a suitable feedback controller that meets your design objectives. A mixed optimization approach based on the Method of Inequality (MOI) offers flexibility when it comes to specifying controller design requirements. For instance, the controller for the system may need to meet certain criteria like achieving a rise time of less than one second, settling within five seconds, and limiting overshoot to under 10%. In such scenarios, it’s evidently more practical and straightforward to express the design problem explicitly using these inequalities. The Method of Inequalities, as described in [42], is a computer-assisted multi-task design methodology that represents desired performance through algebraic inequalities. The primary objective of the control process satisfy the inequalities.

### 3.4 *H*_∞_ Model-based mixed optimization

**Fig 7:**
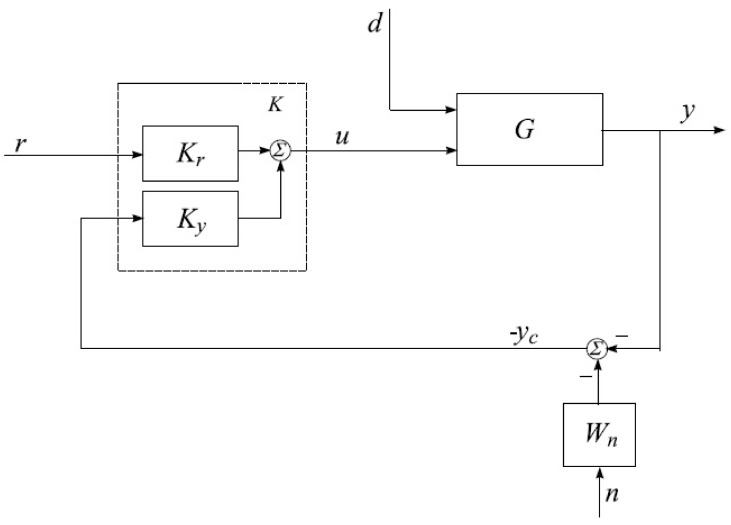
Block diagram of input/output scheme.

The control system’s specification involves tracking a desired signal, as illustrated in the block diagram with the input-output scheme depicted in Fig 7. The objective of the designed control system is to ensure that its output closely follows the predetermined signal. Given the nature of the nonlinear system, it is imperative to employ efficient optimization techniques, as discussed in [43, 44].

**Fig 8:**
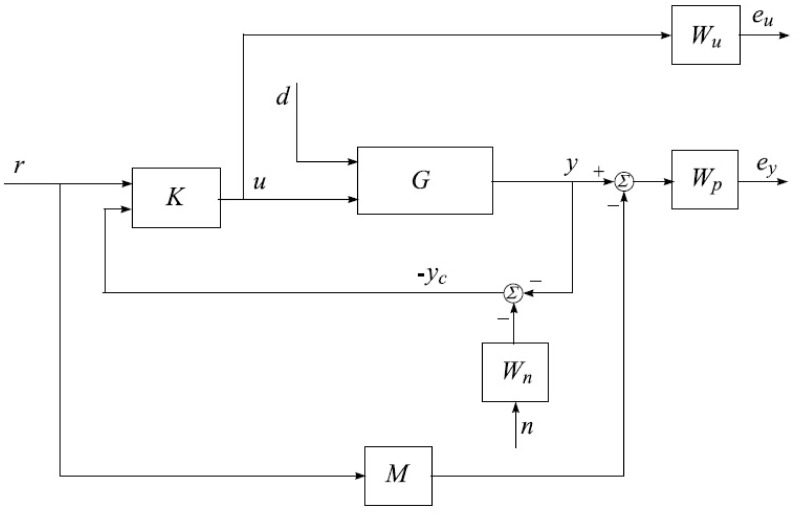
Closed-loop system with performance requirements.

**Fig 9:**
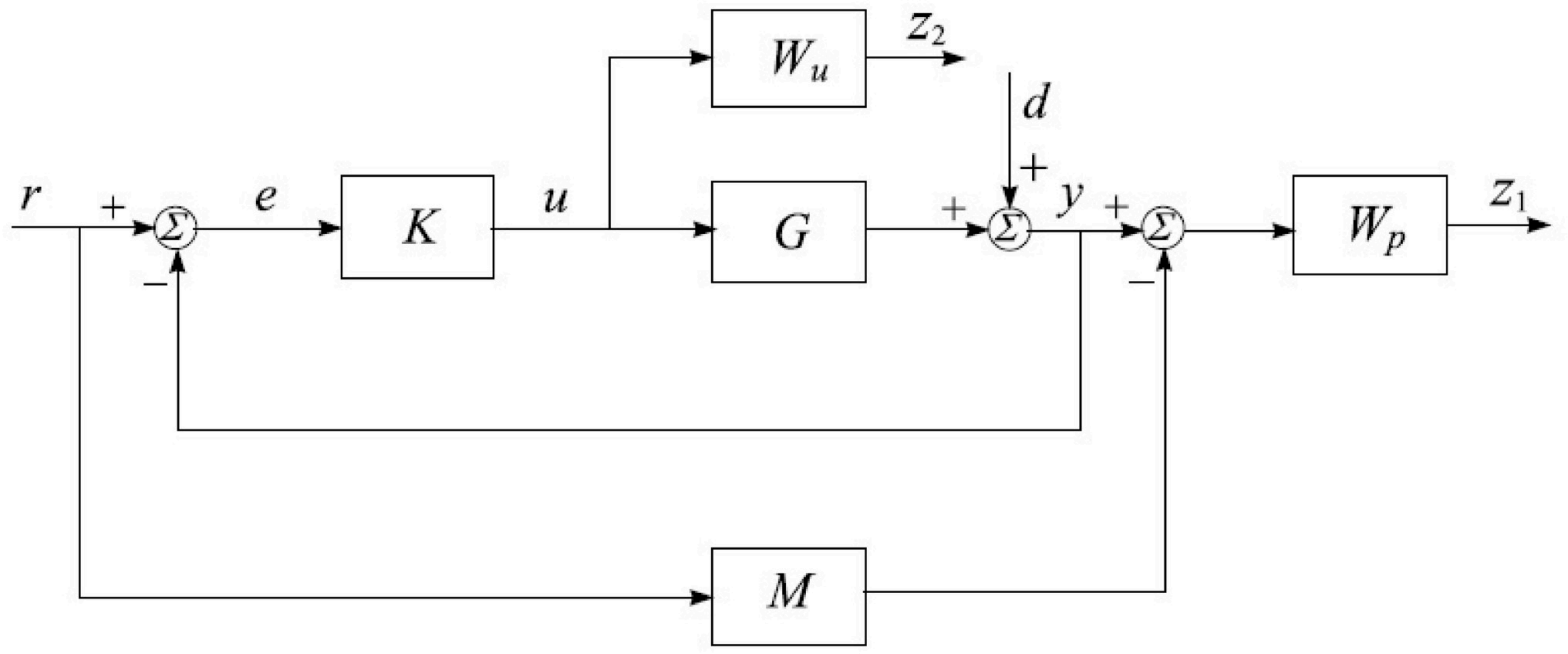
Model based control strategy.

*H*_∞_ model-based mixed optimization offers robustness, versatility, and flexibility when designing control systems for higher-order UAVs with coupling effect. Its ability to handle uncertainties, consider both frequency and time-domain performance, and adapt to system variations makes it a suitable choice for complex UAV applications. By finding an optimal trade-off between performance and robustness, the proposed controller provides a reliable control solution for higher-order UAV systems. Through this approach, two controllers are formulated. The first one focuses on achieving robust stability, internal stability, and disturbance rejection, as depicted in Fig 8. The second controller is designed with the flexibility to use the weighting matrix (tuning parameters) of mixed optimization as a design parameter, guided by the MOI. The term “uncertain plant” refers to the controlled system, which exhibits behavior influenced by uncertainties, variations, and disturbances. When designing a controller for the uncertain plant, some basic requirements must be fulfilled to ensure stable and robust performance, especially in the presence of perturbations. The uncertain plant must fulfill fundamental criteria in the presence of perturbations, including both internal and external disturbances.

$$\begin{array}{c} u=[\begin{array}{cc} K_{r} & K_{y} \end{array}]{\left[\begin{array}{cc} r & -y_{c} \end{array}\right]}^{T}=K_{r}r-K_{y}y_{c} \end{array}$$ (29)

where *K*_*y*_ represents the feedback matrix function and *K*_*r*_ is the transfer function matrix of the pre-filter. The control input signal (*u*) is the signal that drives the UAV to achieve the desired output. It is determined by the error signals (*r*) and *y*_*c*_ through the controller matrix [*Kr Ky*]. The error signal (*r*) represents the difference between the desired reference input and the actual output of the UAV. The other error signal *y*_*c*_ is derived from the output of the UAV and is typically used in feedback control systems. The controller matrix [*K*_*r*_ *K*_*y*_] combines two sub-matrices *Kr* and *Ky*. *Kr* is the transfer function matrix of the pre-filter, and *Ky* is the feedback matrix function. This matrix relates the error signals to the control input *u*, shaping how the errors affect the control action. In Eq (27), the control input (*u*) is equal to the product of the controller matrix [*K*_*r*_ *K*_*y*_] and the column vector [*r*; −*y*_*c*_]. The negative sign in front of *y*_*c*_ indicates that the feedback is used in a negative sense (subtraction) in the control loop. The term [*K*_*r*_ *r*] represents the control action based on the reference input *r*, and it is called the pre-filter. The pre-filter modifies the reference input to shape the desired behavior and compensate for any disturbances that may be present. The term [*K*_*y*_ *y*_*c*_] represents the control action based on the feedback signal *y*_*c*_. The feedback control is used to correct any discrepancies between the desired output and the actual output of the plant. By combining the pre-filter and feedback control, the controller [*K*_*r*_ *K*_*y*_] can effectively regulate the uncertain plant to achieve the desired output while mitigating the effects of internal and external disturbances. Fig 9 depicts the closed-loop model, which encompasses the uncertain TRMS. This model incorporates the feedback response of the controller, performance criteria, and the disturbance matrix associated with noise functions. Within this framework, the variables *r*, *d*, and *n* represent the reference input, input disturbances, and noise, respectively. The goal is to regulate (measure) the output angles, specifically the yaw angle *ϕ* and the pitch angle *θ*.” The aim is to effectively handle various forms of disturbances, such as noise and parametric variations.

The signals for output tracking control, specifically *e*_*y*_ and *e*_*u*_, serve as error tracking signals. The output feedback vector, denoted as *y*_*c*_ and comprising the measured noise *n* and the noise-shaping filter *W*_*n*_, is a vector-matrix. These signals, *e*_*y*_ and *e*_*u*_,” quantify the discrepancies between the desired output and the actual output of the controlled system. These error signals are crucial in closed-loop control as they drive the controller to take corrective actions and regulate the system to track the desired reference input accurately. In a closed-loop control system, the controller compares the reference input with the measured output and calculates the error signals. The control system aims to minimize these error signals to achieve accurate output tracking, which is essential for stable and reliable closed-loop operation. The output feedback vector, denoted as *y*_*c*_, is a vector-matrix that combines the measured output *y* with the measured noise *n*, and it is modified by the filter *W*_*n*_ for noise shaping. The output feedback vector is used in the control loop to make control decisions based on the available measurements and to account for any disturbances or noise present in the system. The output feedback vector *y*_*c*_ can be expressed as: *y*_*c*_ = *y* + *W*_*nn*_. While *y* is the measured output of the controlled system. *W*_*nn*_ represents the noise filter that processes the measured noise *n*. In the context of closed-loop stability, the output feedback vector plays a critical role in ensuring that the control system can handle disturbances, noise, and uncertainties effectively. By incorporating the measured noise and using a noise filter, the control system can shape the control action to minimize the impact of noise on the output and maintain stability and accuracy in the closed-loop system. The following weighted system are required error tracking output (*e*_*y*_, *e*_*u*_) equation must satisfy the condition,

$$\begin{array}{c} \left[\begin{array}{c} e_{y}\\ e_{u} \end{array}]=[\begin{array}{ccc} W_{p}(S_{o}G_{u}K_{r}-M) & W_{p}S_{o}G_{d} & -W_{p}S_{o}G_{u}K_{y}W_{n}\\ W_{u}S_{i}K_{r} & -W_{u}S_{i}K_{y}G_{d} & -W_{u}S_{i}K_{y}W_{n} \end{array}][\begin{array}{c} \begin{array}{c} r\\ d \end{array}\\ n \end{array}\right] \end{array}$$ (30)

while *S*_*i*_ = (*I* + *K*_*y*_*G*_*u*_) − 1 and *S*_*o*_ = (*I* + *G*_*u*_*K*_*y*_) − 1, denote the sensitivity matrix functions for input and output, respectively.

The performance criterion mandates that the transfer function, which relates the external input signals *r*, *d*, and *n* to the signals *e*_*y*_ and *e*_*u*_, must remain small for all potential uncertain plant models denoted as *G*. To accommodate the varying importance of different frequency ranges in achieving these performance criteria, the transfer function matrices *W*_*p*_ and *W*_*u*_ are utilized. These matrices play a role in constructing the transfer function matrix that connects the inputs and outputs within the extended system, as detailed in Table 3. The primary objective of controller design is to regulate the desired output as specified.

$$\begin{array}{c} K=[K_{r}\;\;\;\;\;\;\;\;\;\;\;\;\;K_{y}] \end{array}$$ (31)

The controller design process should be detailed and should ensure compliance with the specified properties in the presence of perturbations. Achieving robust stability in the face of perturbations is essential and involves satisfying both nominal performance and robust response conditions in the closed-loop system. Regarding nominal performance, the conditions to be met include:

$$\begin{array}{c} {\left[\begin{array}{ccc} W_{p}(S_{o,nom}G_{u,nom}K_{r}-M) & W_{p}S_{o,nom}G_{d,nom} & -W_{p}S_{o,nom}G_{u,nom}K_{y}W_{n}\\ W_{u}S_{i,nom}K_{r} & -W_{u}S_{i,nom}K_{y}G_{d,nom} & -W_{u}S_{i,nom}K_{y}W_{n} \end{array}\right]}_{\infty }<1 \end{array}$$ (32)

The condition for the robust performance,

$$\begin{array}{c} {\left[\begin{array}{ccc} W_{p}(S_{o}G_{u}K_{r}-M) & W_{p}S_{o}G_{d} & -W_{p}S_{o}G_{u}K_{y}W_{n}\\ W_{u}S_{i}K_{r} & -W_{u}S_{i}K_{y}G_{d} & -W_{u}S_{i}K_{y}W_{n} \end{array}\right]}_{\infty }<1 \end{array}$$ (33)

These requirements outlined previously must be satisfied for the plant model *G*. The weighting functions, namely, *W*_*p*_ and *W*_*u*_,” are instrumental in establishing the equilibrium or the trade-off among different signal characteristics. Furthermore, the functions *K*_*r*_ and *K*_*y*_ represent the transfer functions of the system matrix and can be easily derived as follows:

$$\begin{array}{c} {}[\frac{\begin{array}{c} z_{1}\\ z_{2} \end{array}}{\begin{array}{c} e_{1}\\ e_{2} \end{array}\;\;}]\;=\;\;\;[\frac{\begin{array}{cc} -W_{p}M & W_{p}\\ 0 & 0 \end{array}\;|\;\begin{array}{c} W_{p}G\\ W_{u} \end{array}}{\;\begin{array}{cc} I & 0\;\;\;\;\;\;\\ 0 & I\;\;\;\;\;\; \end{array}|\;\;\;\begin{array}{c} 0\\ G \end{array}}][\frac{\begin{array}{c} r\\ d \end{array}}{u\;\;}]\; \end{array}$$

The transfer function of the system can be derived as follows:

$$\begin{array}{c} T_{zw}=[\begin{array}{cc} W_{p}(S_{o}G_{u}K_{r}-M) & W_{p}S_{o}\\ W_{u}S_{i}K_{r} & W_{p}K_{y}S_{o} \end{array}] \end{array}$$ (34)

The primary task of the strategy is to overcome the value of the cost function, specifically the *H*_∞_ norm of *T*_*zw*_, while ensuring the gain remains stable. The choice of weighting functions is critical in shaping the system’s response to meet the desired output requirements, as specified in Eq (33).

$$\begin{array}{c} w_{m}=[\begin{array}{cc} w_{m_{11}} & w_{m_{12}}\\ w_{m_{21}} & w_{m_{22}} \end{array}] \end{array}$$ (35)

A model matrix is a mathematical representation of the dynamics of a control system. In the context of the twin-rotor MIMO system, the model matrix would describe the physical behavior of the system, including the interactions between the two rotors and any external disturbances. For an ideal model, the model matrix would accurately capture all the relevant dynamics of the twin-rotor MIMO system. This would include the aerodynamic effects on the rotors, the interactions between the rotors, and any external disturbances that may affect the system. An ideal model would also accurately capture the nonlinearities and uncertainties of the system. A performance matrix is a mathematical representation of the desired performance specifications for a control system. It is typically used in the design of feedback controllers to ensure that the system meets certain performance requirements. A performance matrix is a mathematical representation of the desired performance specifications for a control system. It is typically used in the design of feedback controllers to ensure that the system meets certain performance requirements. The performance matrix is represented in Eq (34).

$$\begin{array}{c} w_{p}=[\begin{array}{cc} w_{p_{11}} & w_{p_{12}}\\ w_{p_{21}} & w_{p_{22}} \end{array}] \end{array}$$ (36)

In the context of a UAV control, the performance matrix would define the desired closed-loop response of the system. This would include specifications such as settling time, overshoot, steady-state error, and bandwidth, among others. The contribution of the performance matrix in the control of a UAV using an *H*_∞_ model-based mixed optimization is that it provides a way to specify the desired performance of the system in a precise and quantitative manner. By defining the performance matrix, the designer can ensure that the controller will meet the desired performance specifications.

**Table 3:** Weighted functions.

Functions	Description
*W*_*p*_(*S*_*o*_*G*_*u*_*K*_*r*_ − *M*)	Weighting functions difference
*W*_*p*_ *S*_*o*_ *G*_*d*_	Weighted function for sensitivity to disturbance
*W*_*p*_ *S*_*o*_ *G*_*u*_ *K*_*y*_ *W*_*n*_	Weighted function for sensitivity to noise
*W*_*u*_ *S*_*i*_ *K*_*r*_	Weighted function for control action regarding reference
*W*_*u*_ *S*_*i*_ *K*_*y*_ *G*_*d*_	Weighted function for control action regarding disturbance
*W*_*u*_ *S*_*i*_ *K*_*y*_ *W*_*n*_	Weighted function for control action regarding noise

A control action matrix is a matrix that maps the measured outputs of the control system to the input signals of the controller. In the context of the UAV, the control action matrix would describe how the controller uses the measurements of the system to generate control signals for the two rotors provided in Eq (35).

$$\begin{array}{c} w_{u}=[\begin{array}{cc} w_{u_{1}} & 0\\ 0 & w_{u_{2}} \end{array}] \end{array}$$ (37)

The control action matrix is an important component of the control system, as it determines how the controller responds to changes in the system. A well-designed control action matrix will enable the controller to respond quickly and accurately to changes in the system, resulting in better control performance. In the control of the UAV using an optimization, the control action matrix is used in conjunction with the model matrix to design a controller that is robust to disturbances and uncertainties. The *H*_∞_ model-based mixed optimization is designed to optimize the control action matrix to achieve the desired performance specifications while maintaining stability in the presence of disturbances and uncertainties.

The objective of the control action matrix in the control of the UAV allows the controller to use the measurements of the system to generate control signals that achieve the desired performance specifications. By designing a control action matrix that is optimized for the specific dynamics of the system, the controller can achieve better performance than a controller that does not use an optimized control action matrix. A sensor noise matrix is a matrix that describes the effect of noise on the measurements taken by the sensors in a control system and the mathematical expression given in Eq (36). In the context of the UAV, the sensor noise matrix would describe the noise characteristics of the sensors that measure the outputs of the system, such as the rotor positions and velocities. The sensor noise matrix is an important component of the control system, as it determines the accuracy and reliability of the measurements used by the controller to make control decisions. A well-designed sensor noise matrix will allow the controller to make accurate control decisions despite the presence of noise in the sensor measurements.

$$\begin{array}{c} w_{n}=[\begin{array}{cc} w_{n_{1}} & 0\\ 0 & w_{n_{2}} \end{array}] \end{array}$$ (38)

**Fig 10:**
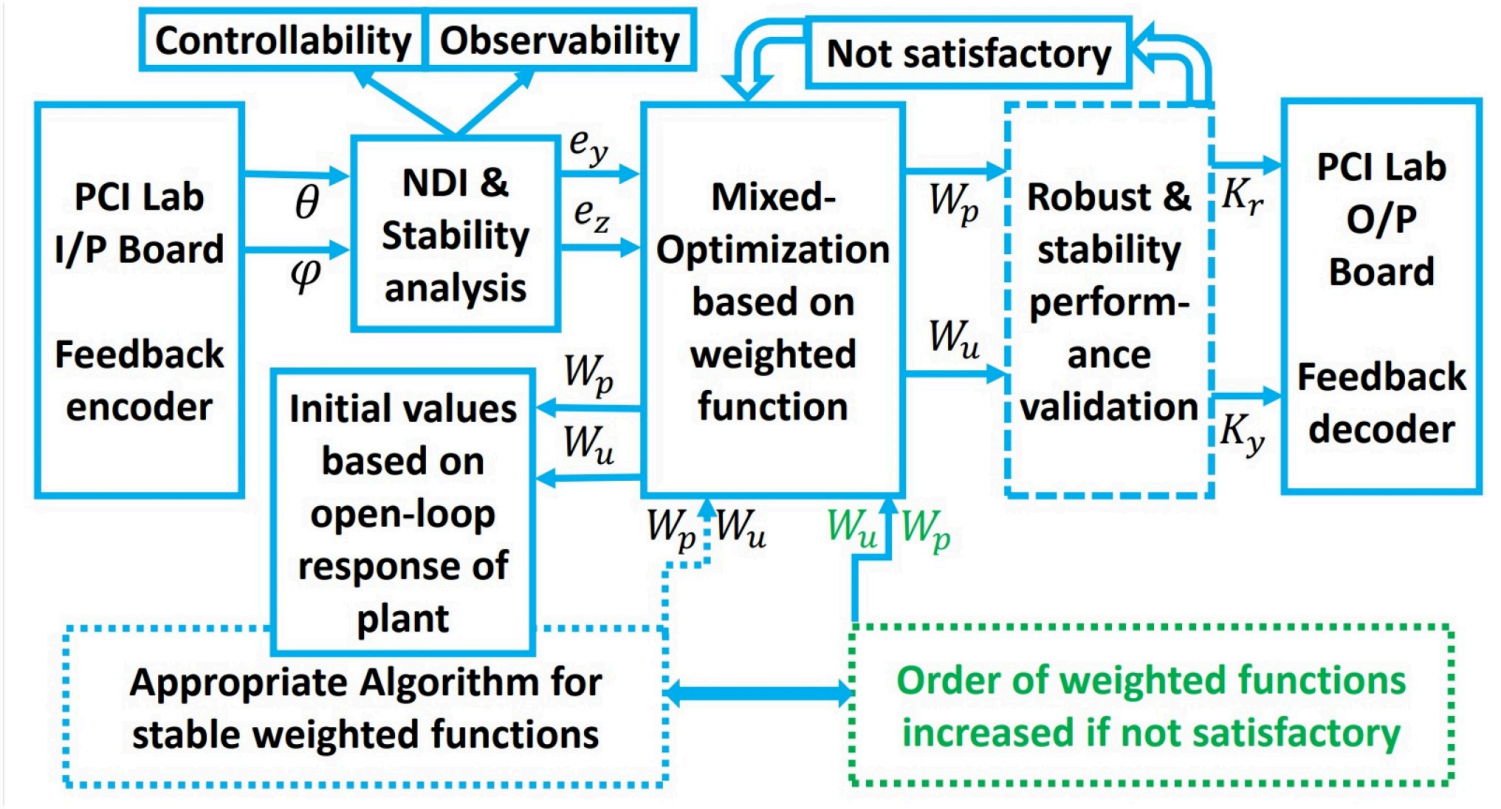
Block diagram of mixed optimization.

In the control of the UAV, the sensor noise matrix is used in conjunction with the model matrix and the control action matrix to design a controller that is robust to disturbances, uncertainties, and sensor noise. The *H*_∞_ Model-based mixed optimization is designed to minimize the effect of sensor noise on the control performance by optimizing the controller gains based on the sensor noise matrix. The contribution of the sensor noise matrix in the control of the UAV using a controller is that it allows the controller to make accurate control decisions despite the presence of sensor noise. By designing a sensor noise matrix that accurately characterizes the noise characteristics of the sensors, the controller can optimize the controller gains to minimize the effect of sensor noise on the control performance. The controller design process, along with its calculation steps, is illustrated in the flowchart presented in Fig 10. If unsatisfactory conditions are encountered for the upper and lower bounds, adjustments in the weighting functions may be necessary. It is imperative to ensure that the cost function value remains below one, indicating the effectiveness of the controller design.

The interconnection of the closed-loop plant, incorporating the weighting matrix, can be visualized by examining the singular value plot presented in the simulation results. The achieved values serve as indicators of the system’s potential for achieving stability and robust performance. In the case of using the *H*_∞_ controller, the calculated gamma value (*γ*) is 0.1006, which is less than one. This suggests that both stability and robust performance can be attained. Throughout the iterative process, exploring gamma (*γ*) values ranging from 0.1 to 10 with a tolerance of 0.001 helps discern which gamma values are acceptable and which are not. Subsequently, the controller design process is executed to determine the gain *K*.

## 4 Simulation results

**Fig 11:**
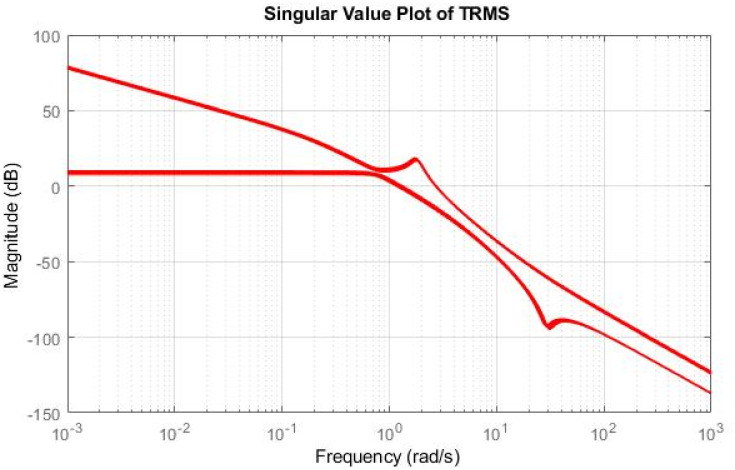
Singular value of open-loop system.

**Fig 12:**
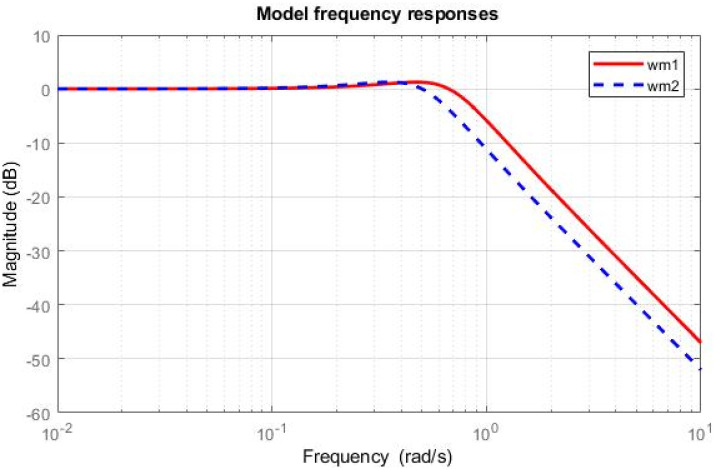
Model frequency response.

**Fig 13:**
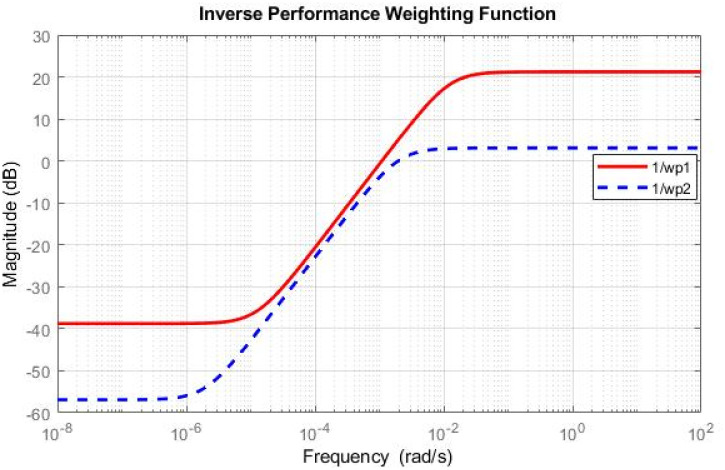
Inverse performance weighted function.

**Fig 14:**
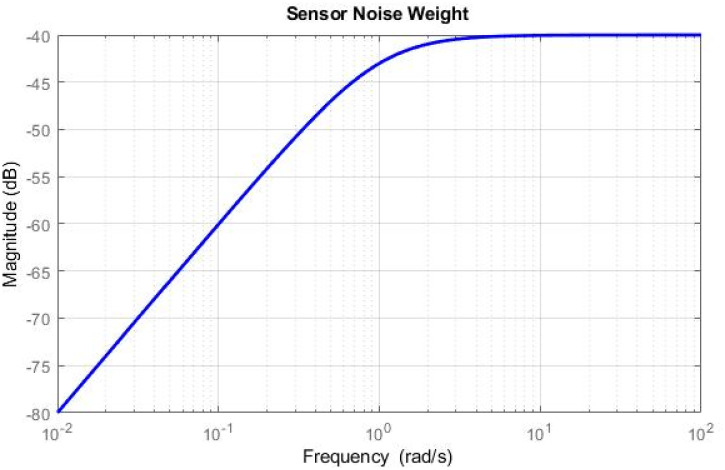
Magnitude of noise frequency for uncertain system.

**Fig 15:**
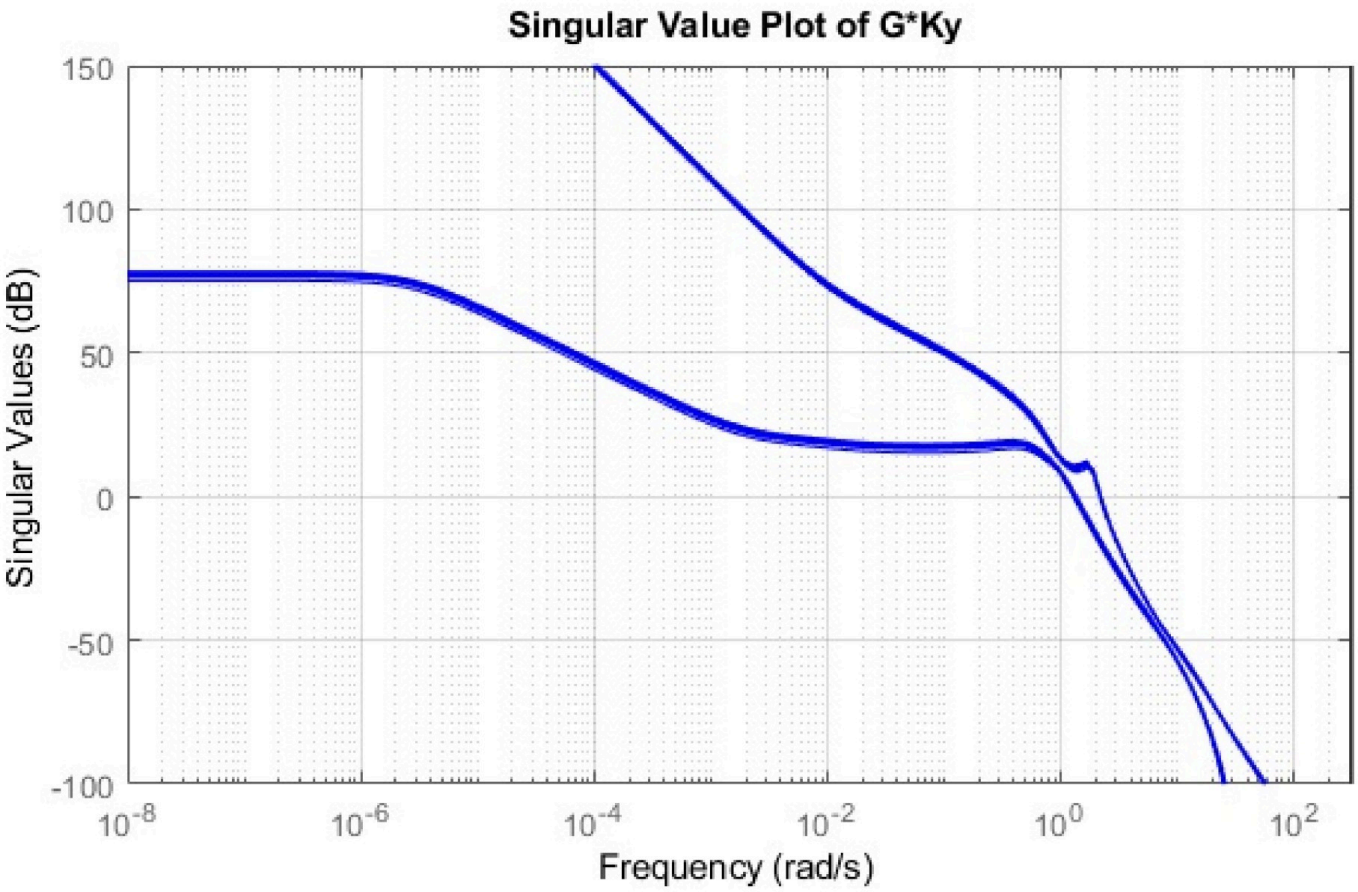
Singular value with gain (G*Ky).

**Fig 16:**
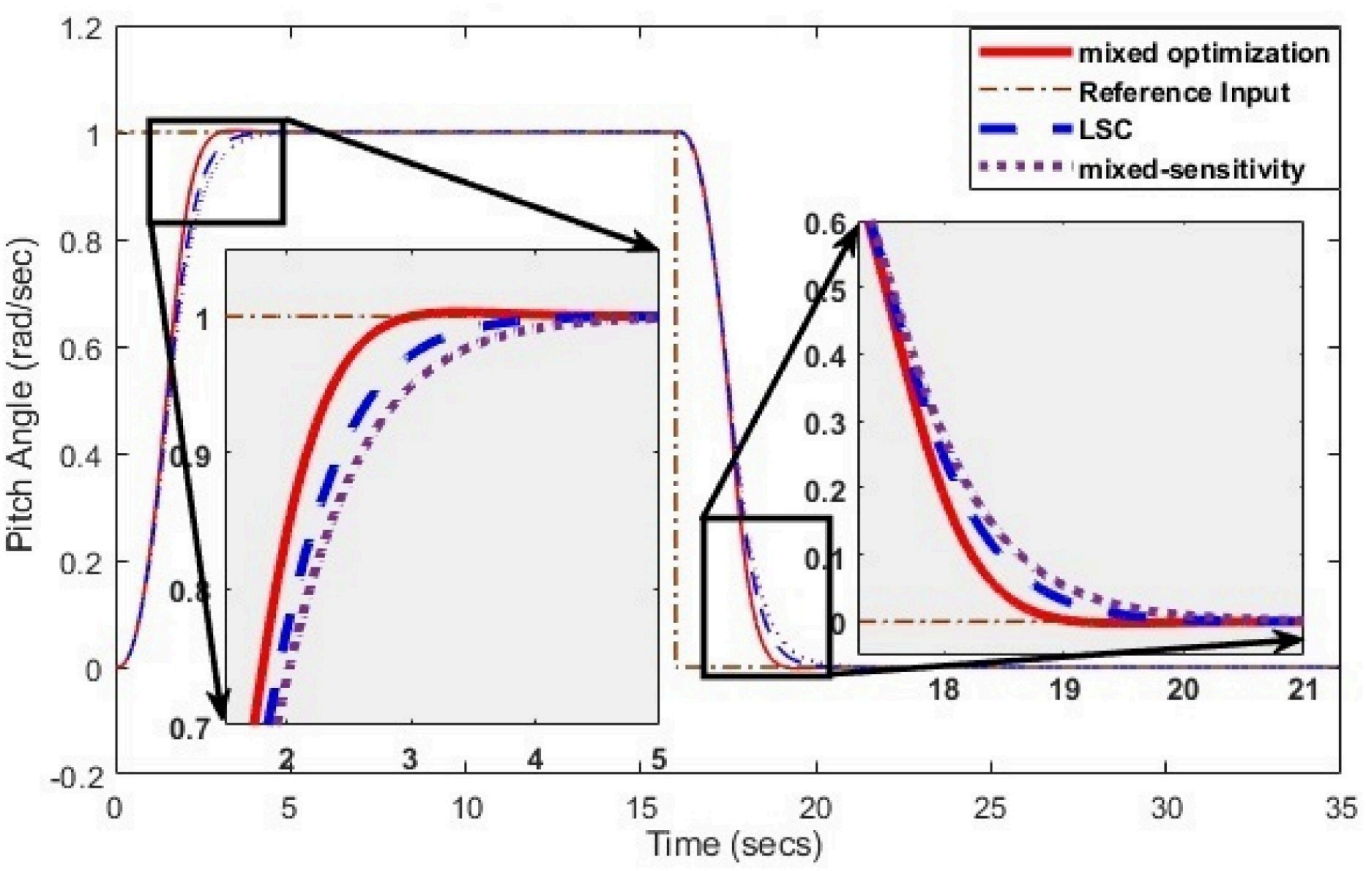
Comparison of optimizations for pitch angle.

**Fig 17:**
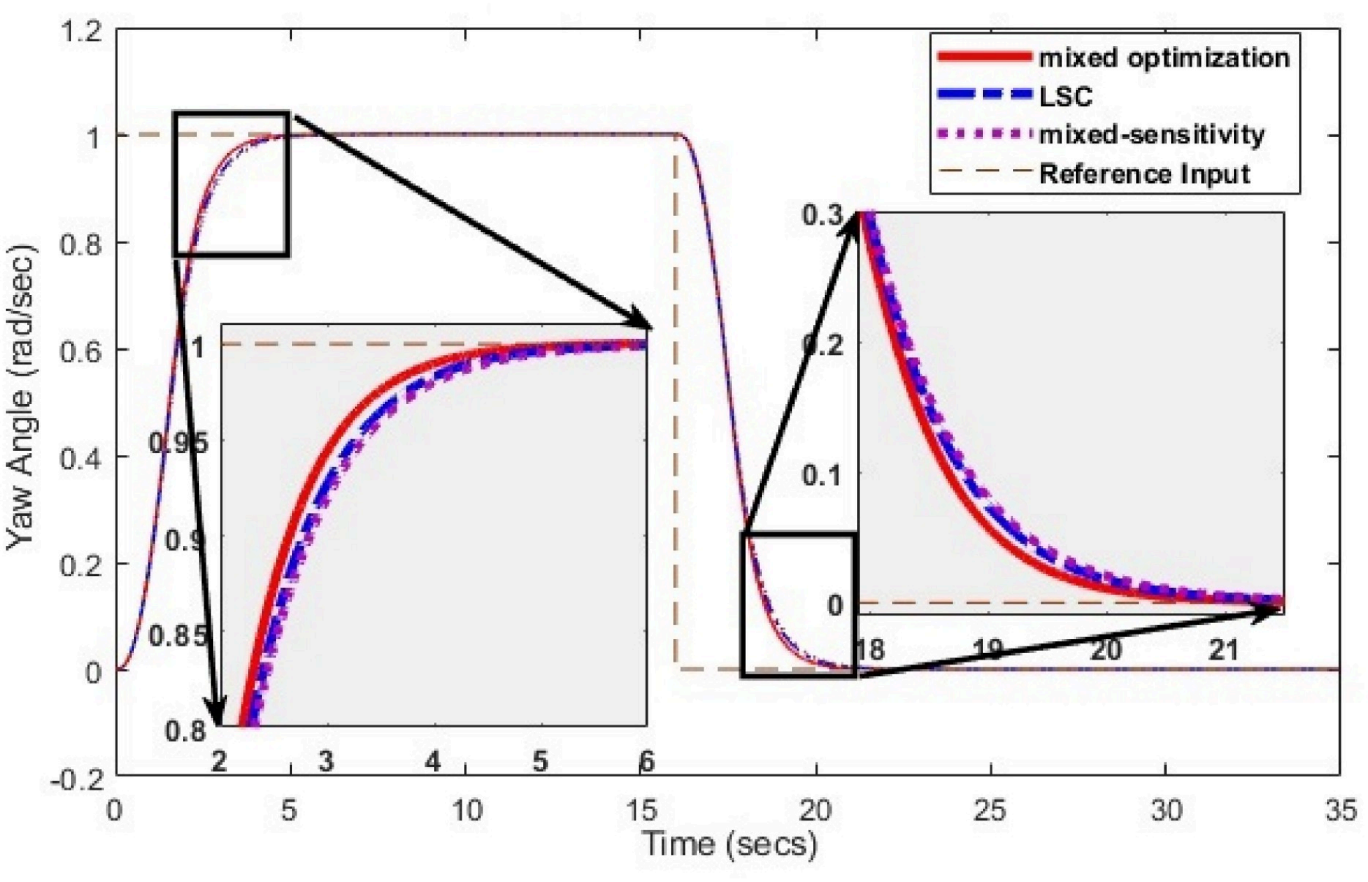
Comparison of optimizations for yaw angle.

Simulations have been conducted to analyze the control behavior of both angles, evaluating the robustness of the implemented techniques concerning matched and unmatched perturbations. These simulations utilize the perturbed model of the TRMS to assess the controller’s performance. The controller synthesis involves multiple iterations with various performance criteria and weighting matrices. Fig 11 displays the singular value plot of the open-loop system for the UAV. The utilization of this controller technique enables the rejection of both matched and unmatched uncertainties. The simulations serve to assess the control behavior of both angles and ascertain the robustness of the implemented technique. The perturbed model of the UAV is utilized for these simulations. The simulation response of the model frequency and inverse response of a UAV system provides valuable information about the system’s gain, frequency range, resonances, phase shift, stability margins, and dynamic behavior in the frequency domain, as shown in Figs 12 and 13 respectively. The transfer function of *w*_*n*_ provides a magnitude frequency response represented in Fig 14. The singular value of the closed-loop system multiplied with mixed optimization controller gain is obtained in Fig 15. The simulation response of the singular values provides valuable information about the closed-loop system’s gain, frequency range, robustness, stability margins, sensitivity, and performance trade-off. This knowledge is crucial for control system design, analysis, and optimization to ensure desired system behavior and performance. A comparative simulation response of applied optimizations for the pitch angle of the highly nonlinear system is shown in Fig 16. It can be realized from the simulation response that the convergence time and settling time of mixed optimization are faster than other optimizations. Beyond the convergence time, robust stability and robust performance of the focused strategy ensure efficient results. The yaw angle of the TRMS also represented in Fig 17 with detailed information on the convergence states. On the basis of sharp convergence and excellent robustness against perturbations (unmodeled dynamics, noise interference, parameter variation, and disturbance signal-like disturbance torque), mixed optimization is best for a higher-order MIMO system. The strategy also rejects the uncertainties as: *dist*(1) = 0.2 and *dist*(2) = 0.1, *noise*(1) = 0.1*andnoise*(2) = 0.1. On the other hand, real-time realization also supports the remarkable worth of the considered optimization.

### 4.1 Experimental setup and results

In this section, we explore the concept of real-time implementation and system interconnections through the use of system-integrated circuits. Additionally, we offer a concise overview of the conclusions drawn from the validated output results.

**Fig 18:**
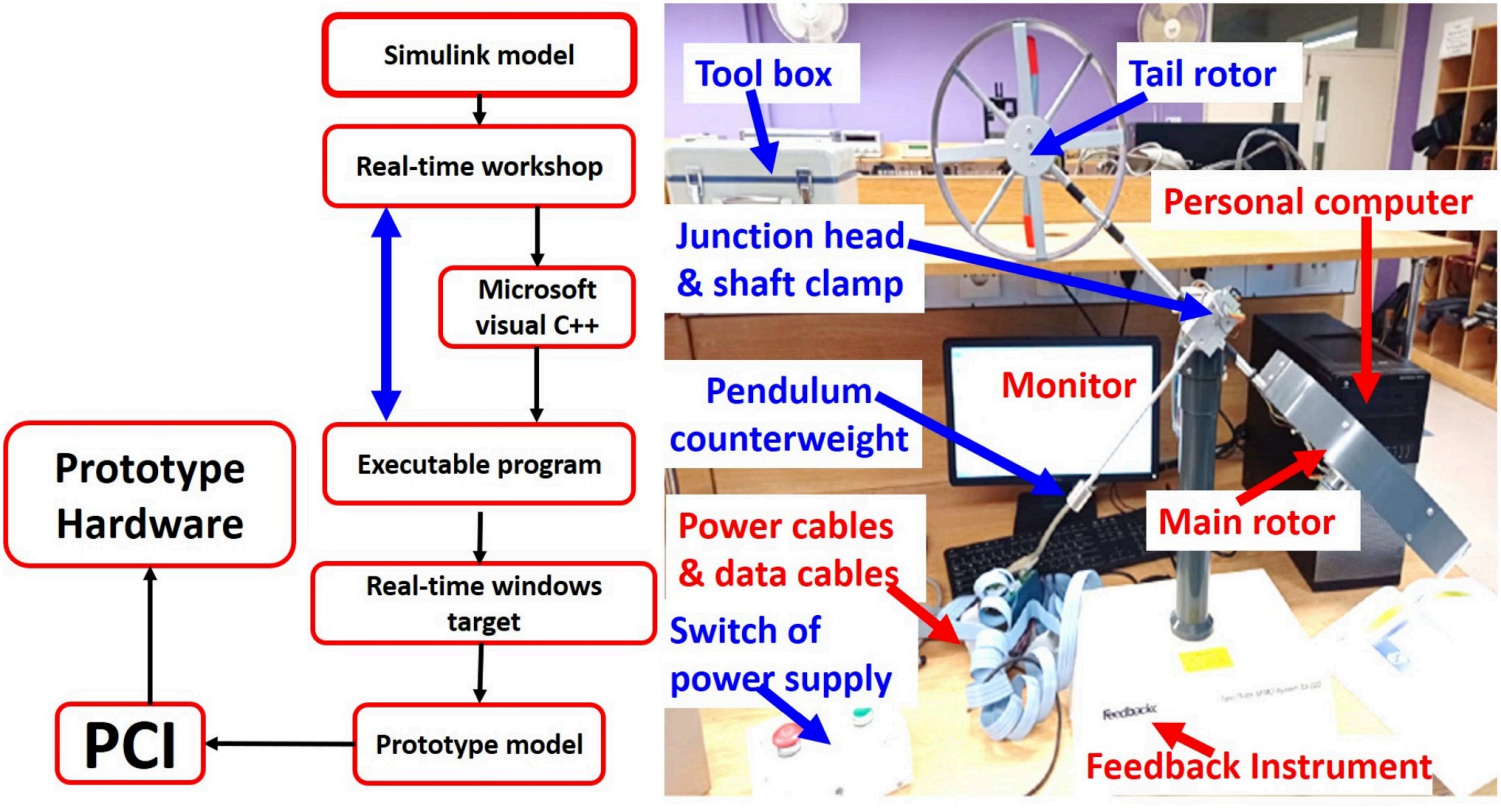
(a) Implementation design steps, (b) Experimental setup.

**Fig 19:**
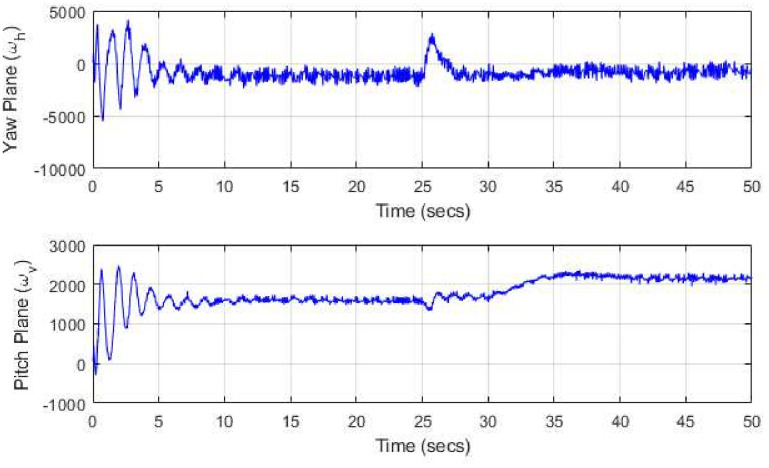
Rotors speed.

The implementation design steps and the experimental setup for the UAV are illustrated in Fig 18a and 18b, respectively. These diagrams offer insights into the laboratory hardware prototype and the design implementation process. The *H*_∞_ model-based mixed optimization, tailored to varying dynamics, is performed with different performance weighting matrices (tuning factors). The control action for both rotors is elucidated through simulation responses. The controller’s application allows for the rejection of unwanted signals and perturbations, including parametric variations, disturbance torque, thrust fluctuations, and external uncertainties. To confirm the robust performance of the model, we expose it to a worst-case scenario, which includes a 10% parametric uncertainty and the real-time application of disturbance noise signals to both rotors. The real-time implementation, facilitated by a robustly designed and flexible nature controller, serves to demonstrate the controller’s reliability in the presence of disturbances such as noise signals, un-modeled states, parametric variations, and coupling effects. The rotor speeds for both motors are depicted in Fig 19.

**Fig 20:**
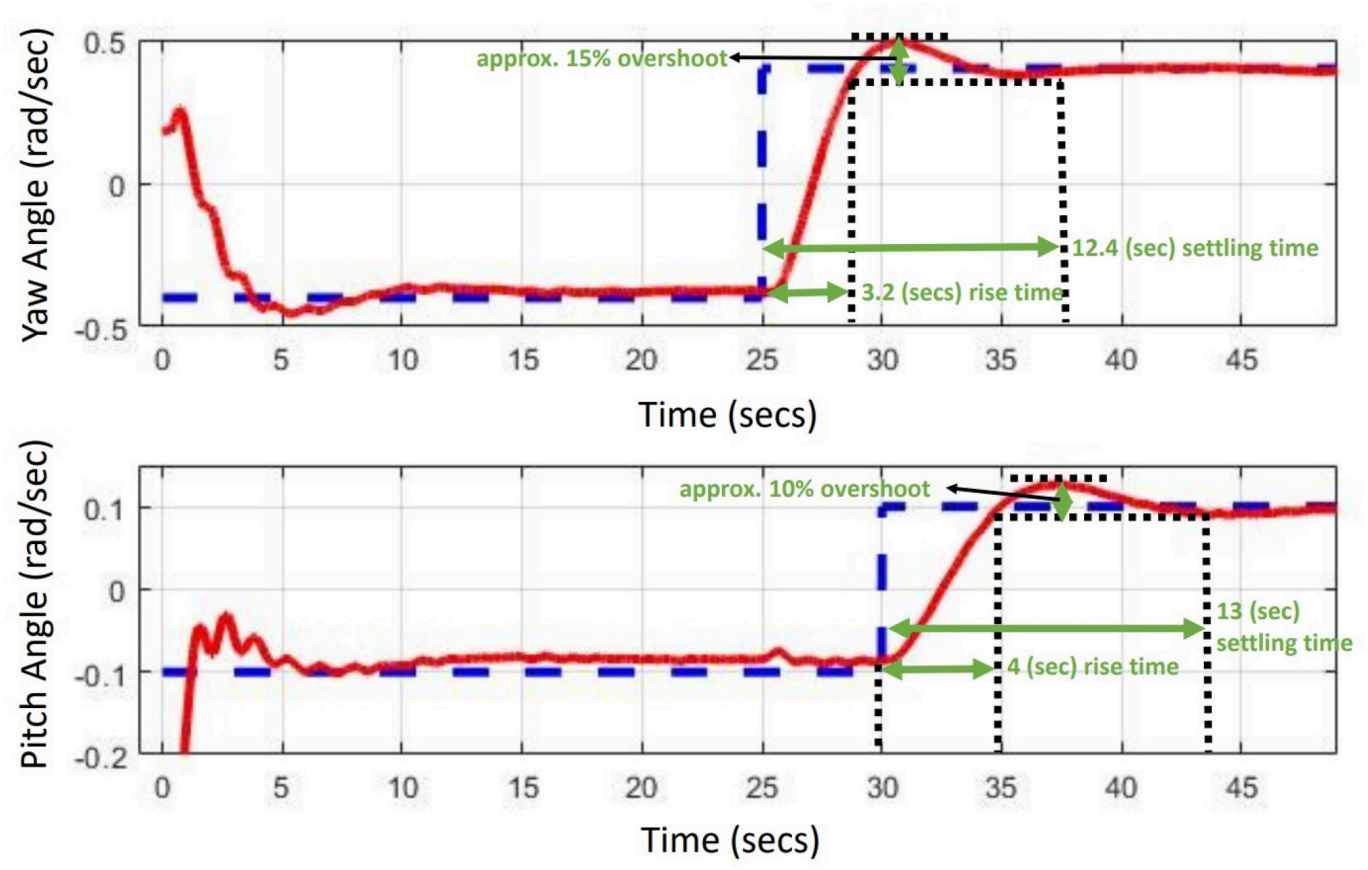
Experimental pitch and yaw angle response of TRMS.

**Fig 21:**
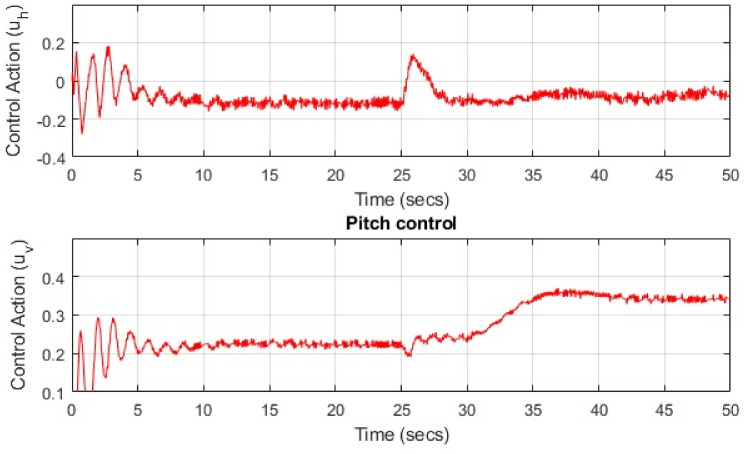
Control input response for TRMS.

Limited perturbations, including parametric variations and wind disturbances, are introduced to assess the system’s robust response and stability, thus affirming the controller’s effectiveness. The experimental output responses for the pitch angle and yaw angle are displayed in Fig 20, providing evidence of the system’s ability to converge within constrained variations while tracking the reference input. The figure depicting the square input for both the pitch angle and yaw angle of the TRMS highlights that the pitch angle experiences a faster convergence compared to the yaw angle. This difference can be attributed to the initial need to stabilize the main rotor to counteract disturbances generated by the tail rotor, including gyroscopic torque effects and coupling effects. Consequently, the convergence time of the tail rotor cannot be as rapid as that of the main rotor. The step input tracking response observed in the simulation validates the control theory related to the convergence time of angles, particularly the pitch and yaw angles, as it yields a sharp stability response. The response of the control actions for both angles can be characterized by minimal voltage variations, underscoring the effectiveness of the proposed controller. The attenuation observed in the simulation response can be ascribed to both the introduced noise signal and the controller’s effective handling of a wide range of disturbances. Furthermore, the results illustrate how the controller gradually mitigates the introduced disturbance as time elapses, enhancing system stability—a distinguishing feature when compared to other control methods. It is noteworthy that the convergence time and the degree of attenuation are more pronounced in the yaw angle. This disparity arises due to the heightened coupling effect resulting from disturbance torque, primarily driven by the mass of the weighted rotor and the main rotor blades. Several factors introduced by the main rotor contribute to a slight but distinct variation in the yaw angle. The control action for both the pitch and yaw angles is illustrated in Fig 21. Based on simulation and experimental response, comparative details for settling time, rise time, and overshoot % are provided in Table 4. Particularly, the yaw angle exhibits less overshoot in its amplitude response in comparison to the pitch angle. This distinction can be attributed to the coupling effect generated by the tail rotor, as elucidated in the second section. Additionally, a noticeable sharp variation can be observed in the control action of the yaw angle. The experimental results underscore that linearized systems yield nearly identical responses to nonlinear system responses. Nevertheless, a significant challenge arises from the high levels of noise (disturbance), which can impact the actuators and the accuracy of the input control signal. To obtain the actual actuator input, a first-order filter based on the Butterworth filter is employed. The quantitative measures of a control system (UAVs) are of great importance in experimental response. They provide insights into system behavior, aid in control design, stability analysis, trajectory planning, response speed, stability, accuracy, and system identification. Understanding these properties is essential for developing effective control strategies and optimizing the system’s performance in real-world applications. A brief detail of current research achievements and proposed method is represented in Table 5.

**Table 4:** Control strategies comparison.

Quantitative/numerical details	*H*_∞_ *LSC*	*H*_∞_ mixed-sensitivity	*H*_∞_ mixed optimization
Control structure flexibility	not good	not good	excellent
Handling of multivariable systems	poor	poor	very good
Settling time pitch angle (*rad*/*s*)	3.5	3.2	2
Settling time yaw angle (*rad*/*s*)	4	3.6	2.5
Overshoot	minor overshoot	minor overshoot	zero overshoot
Robust against parametric uncertainties	not good	not good	yes
Robust against perturbations.	not satisfactory	not satisfactory	satisfactory
Real-time implementation robustness	poor response	poor response	satisfactory
Experimental response details of *H*_∞_ mixed optimization	Settling time (*s*)	Rise time (*s*)	Overshoot %
Pitch angle (*rad*/*s*)	13	4	10
Yaw angle (*rad*/*s*)	12.4	3.2	15

**Table 5:** Experimental response time specifications with control strategies comparison.

Methodology	Settling time (*θ*/*ϕ*)	Rise time (*θ*/*ϕ*)	Overshoot % (*θ*/*ϕ*)	Robustness
Fractional order PID [45]	19.97/17.77	5.87/8.79	38.03/37.65	low
Model free control [46]	15.45/12.17	3.51/1.97	34.62/31.85	moderate
Iterative learning control [47]	18.76/16.25	8.36/5.23	20.13/17.69	high
Proposed algorithm	13.16/12.43	4.17/3.23	2.10/2.59	very high

#### 4.1.1 Remark

The mixed optimization with *H*_∞_ model-based control approach offers robustness, decoupling, and performance trade-offs and permits handling model uncertainty while ensuring stability for a highly coupled TRMS. The weighted functions augmentation with *H*_∞_ model-based control as a mixed optimization provides combined benefits. The flexible approach in terms of the design parameters makes it a fully developed algorithm to guarantee stability and robust performance under perturbations (external and internal disturbances with unstructured dynamics of the MIMO system). These advantages make the proposed mixed control approach a flexible and suitable choice for controlling complex MIMO systems like TRMS.

## 5 Conclusion

This paper represents the culmination of multiple attempts to develop a performance controller that is robust and stable for a prototype helicopter model, referred to as the UAV. This model is highly nonlinear and exhibits a significant coupling effect between its pitch and yaw rotors, both of which are controlled by individual DC motors. The design of an appropriate controller to achieve robust optimization for the UAV has proven to be a challenging undertaking. To derive its mathematical model, certain assumptions had to be made due to the nonlinearities present in the system. The controller design process involved two phases. The project proceeded in two main phases. In the initial phase, we linearized the system using the NDI (Nonlinear Dynamic Inversion) process, and assessed its stability by performing controllability and observability matrix calculations. In the subsequent phase, we employed an H-infinity model-based control approach, enhanced with mixed optimization control techniques, to attain the desired optimized system response.

The design challenge was framed by utilizing weighting function parameters as the key design variables to fulfill a set of closed-loop performance constraints. This MOI-based mixed optimization approach offered greater flexibility in shaping controller design specifications. Notably, it removed the requirement for the designer to specify the controller’s order, instead enabling them to determine the order of the weighting functions. This approach enabled the synthesis of high-order controllers using low-order weighting functions, leading to substantial enhancements in performance and robustness compared to simple low-order controllers. To achieve robust control, the weights were iteratively selected to achieve high gains for low frequencies and low gains for high frequencies. These weight selections were based on stability and robustness performance results obtained through simulations. The controllers were implemented using the Simulink/MATLAB environment. Real-time implementation of the *H*_∞_ model-based mixed optimization approach was carried out to verify the controller’s robust behavior in the presence of both matched perturbations (such as parametric and coupling effects) and mismatched perturbations (such as wind disturbances) The quantitative measures of a control system (UAVs) in experimental response are also focused on. The experimental results demonstrate a significant improvement in the robust control of the UAV, with a reduction in steady-state error by 35% and a decrease in overshoot by 25%. Additionally, the proposed control approach achieved a 15% increase in the system’s settling time, indicating faster response and enhanced stability. Several recommendations for control engineers have been derived from the analysis of the experimental findings provided earlier.

- Achieving complete elimination of disturbance torque from the tail rotor using the model matrix is unattainable in real-time performance evaluation.
- The experimental confirmation of the effectiveness of robust control optimization reveals that the TRMS behavior during real-time implementation is highly sensitive to precise tuning and the choice of weighting functions (tuning parameters).
- Real-time performance analysis indicates that it is impossible to entirely eliminate the disturbance torque of the tail rotor through the decoupling process.
- The presence of a high-amplitude noise signal results in significant interference with the input actuators and introduces high-frequency components.
